# Supplementary material for: FIRST-line support for assistance in breathing in children (FIRST-ABC): a master protocol of two randomised trials to evaluate the non-inferiority of high-flow nasal cannula (HFNC) versus continuous positive airway pressure (CPAP) for non-invasive respiratory support in paediatric critical care
Source: BMJ Open. 2020 Aug 4;10(8):e038002. doi: 10.1136/bmjopen-2020-038002 (PMC7406113; doi:10.1136/bmjopen-2020-038002)
Supplement: Supplementary data [file bmjopen-2020-038002supp001.pdf]

To be printed on local hospital headed paper

icnarc intensive care national audit & research centre — FIRST ABC — Great Ormond Street Hospital for Children NHS Foundation Trust

FIRST-line support for Assistance in Breathing in Children

Consent Form - Parent or Legal Guardian

Version 1.2, 17 January 2020

To be completed by the Researcher:

Hospital name:	
Trial Number:	
Child's full name:	

To be completed by the Parent or Legal Guardian:

Once you have read and understood each statement – **if you agree, please write your initials in each box**

1.	I confirm that I have read and understood the Participant Information Sheet (version 1.2, dated 27/11/2019) for the above research study. I have had the opportunity to consider the information, ask questions and have had these answered satisfactorily.	☐
2.	I understand that participation is voluntary, and that I am free to withdraw consent at any time, without giving any reason and without my child's medical care or legal rights being affected.	☐
3.	I agree to for my child to continue to take part in this study.	☐
4.	I understand that relevant sections of my child's medical records and data collected during the study (including name, date of birth, postcode and NHS number), held by the NHS or by NHS Digital, may be looked at by individuals from the NHS Trust, the Intensive Care National Audit & Research Centre (ICNARC), NHS Digital or regulatory authorities where it is relevant to my participation in this research. I give permission for these individuals to have access to my child's records.	☐
5.	I agree to complete a questionnaire about my experiences and reactions to being in the intensive care or high dependency unit.	☐
6.	I understand that ICNARC will send me a questionnaire to find out how my child is doing in six months time.	☐
7.	I understand that the information collected in the study will be used to support other research in the future, and may be shared anonymously with other researchers.	☐
8.	I would like to be contacted about any future related studies.	☐

Your signature:		Date:	
Your full name (PRINT):			
Researcher signature:		Date:	
Researcher full name (PRINT):			

Once signed <u>please turn over</u> and complete

Richards-Belle A, *et al. BMJ Open* 2020; 10:e038002. doi: 10.1136/bmjopen-2020-038002

FIRST-line support for Assistance in Breathing in Children

Parent or Legal Guardian contact information

To be completed by the Parent or Legal Guardian:

If you would prefer to receive the questionnaire (as detailed on point 6 of the Consent Form) by **email**, please provide your details below:

**Email address:**	
**Telephone number(s):**	

OR

if you would prefer to receive the questionnaire in the **post**, please provide your details below:

**Postal address:**								
**Postcode:**								
**Telephone number(s):**								

1 copy for patient and parent/guardian; 1 copy for Investigator Site File; 1 copy to be kept with hospital notes

Richards-Belle A, *et al. BMJ Open* 2020; 10:e038002. doi: 10.1136/bmjopen-2020-038002
