# Supplementary material for: FIRST-line support for assistance in breathing in children (FIRST-ABC): a master protocol of two randomised trials to evaluate the non-inferiority of high-flow nasal cannula (HFNC) versus continuous positive airway pressure (CPAP) for non-invasive respiratory support in paediatric critical care
Source: BMJ Open. 2020 Aug 4;10(8):e038002. doi: 10.1136/bmjopen-2020-038002 (PMC7406113; doi:10.1136/bmjopen-2020-038002)
Supplement: Author's manuscript [file bmjopen-2020-038002.draft_revisions.pdf]

BMJ Open is committed to open peer review. As part of this commitment we make the peer review history of every article we publish publicly available.

When an article is published we post the peer reviewers' comments and the authors' responses online. We also post the versions of the paper that were used during peer review. These are the versions that the peer review comments apply to.

The versions of the paper that follow are the versions that were submitted during the peer review process. They are not the versions of record or the final published versions. They should not be cited or distributed as the published version of this manuscript.

BMJ Open is an open access journal and the full, final, typeset and author-corrected version of record of the manuscript is available on our site with no access controls, subscription charges or pay-per-view fees (http://bmjopen.bmj.com).

If you have any questions on BMJ Open's open peer review process please email info.bmjopen@bmj.com

BMJ Open

FIRST-line support for Assistance in Breathing in Children (FIRST-ABC): A master protocol of two randomised trials to evaluate the non-inferiority of high flow nasal cannula (HFNC) versus continuous positive airway pressure (CPAP) for non-invasive respiratory support in paediatric critical care

Journal:	*BMJ Open*
Manuscript ID	bmjopen-2020-038002
Article Type:	Protocol
Date Submitted by the Author:	24-Feb-2020
Complete List of Authors:	Richards-Belle, Alvin; Intensive Care National Audit and Research Centre, Clinical Trials Unit<br>Davis, Peter; Bristol Royal Hospital for Children, Paediatric Intensive Care<br>Drikite, Laura; Intensive Care National Audit and Research Centre, Clinical Trials Unit<br>Feltbower, Richard; University of Leeds, Leeds Institute for Data Analytics<br>Grieve, Richard; London School of Hygiene & Tropical Medicine, Department of Health Services Research and Policy<br>Harrison, David; Intensive Care National Audit and Research Centre, Clinical Trials Unit<br>Lester, Julie; Parent representative<br>Morris, Kevin; Birmingham Women's and Children's Hospitals NHS Foundation Trust, Paediatric Intensive Care Unit<br>Mouncey, Paul; Intensive Care National Audit and Research Centre, Clinical Trials Unit<br>Peters, Mark; Great Ormond Street Hospital For Children NHS Trust, Paediatric Intensive Care Unit; University College London, Institute of Child Health<br>Rowan, Kathryn; Intensive Care National Audit and Research Centre, Clinical Trials Unit<br>Sadique, Zia; London School of Hygiene & Tropical Medicine, Department of Health Services Research and Policy<br>Tume, Lyvonne; University of Salford, School of Health and Society<br>Ramnarayan, Padmanabhan; Great Ormond Street Hospital For Children NHS Trust, Children's Acute Transport Service
Keywords:	Paediatric intensive & critical care < ANAESTHETICS, Paediatric intensive & critical care < INTENSIVE & CRITICAL CARE, Paediatric intensive & critical care < PAEDIATRICS, Clinical trials < THERAPEUTICS, STATISTICS & RESEARCH METHODS

SCHOLARONE™
Manuscripts

I, the Submitting Author has the right to grant and does grant on behalf of all authors of the Work (as defined in the below author licence), an exclusive licence and/or a non-exclusive licence for contributions from authors who are: i) UK Crown employees; ii) where BMJ has agreed a CC-BY licence shall apply, and/or iii) in accordance with the terms applicable for US Federal Government officers or employees acting as part of their official duties; on a worldwide, perpetual, irrevocable, royalty-free basis to BMJ Publishing Group Ltd ("BMJ") its licensees and where the relevant Journal is co-owned by BMJ to the co-owners of the Journal, to publish the Work in this journal and any other BMJ products and to exploit all rights, as set out in our licence.

The Submitting Author accepts and understands that any supply made under these terms is made by BMJ to the Submitting Author unless you are acting as an employee on behalf of your employer or a postgraduate student of an affiliated institution which is paying any applicable article publishing charge ("APC") for Open Access articles. Where the Submitting Author wishes to make the Work available on an Open Access basis (and intends to pay the relevant APC), the terms of reuse of such Open Access shall be governed by a Creative Commons licence – details of these licences and which Creative Commons licence will apply to this Work are set out in our licence referred to above.

Other than as permitted in any relevant BMJ Author's Self Archiving Policies, I confirm this Work has not been accepted for publication elsewhere, is not being considered for publication elsewhere and does not duplicate material already published. I confirm all authors consent to publication of this Work and authorise the granting of this licence.

For peer review only

FIRST-line support for Assistance in Breathing in Children (FIRST-ABC): A master protocol of two randomised trials to evaluate the non-inferiority of high flow nasal cannula (HFNC) versus continuous positive airway pressure (CPAP) for non-invasive respiratory support in paediatric critical care

AUTHORS

Alvin Richards-Belle[1], Peter Davis[2], Laura Drikite[1], Richard Feltbower[3], Richard D. Grieve[4], David A. Harrison[1], Julie Lester[5], Kevin P. Morris[6], Paul R. Mouncey[1], Mark J. Peters[7,8,10], Kathryn M. Rowan[1], M. Zia Sadique[4], Lyvonne N. Tume[9], Padmanabhan Ramnarayan[10,11]

Affiliations

[1]Clinical Trials Unit, Intensive Care National Audit & Research Centre (ICNARC), Napier House, 24 High Holborn, London, WC1V 6AZ, United Kingdom

[2]Paediatric Intensive Care, Bristol Royal Hospital for Children, University Hospitals Bristol NHS Foundation Trust, Upper Maudlin Street, Bristol, BS2 8BJ, United Kingdom

[3]Leeds Institute for Data Analytics, University of Leeds, School of Medicine, Worsley Building, Leeds, LS2 9JT, United Kingdom

[4]Department of Health Services Research and Policy, London School of Hygiene & Tropical Medicine, 15-17 Tavistock Place, London, WC1H 9SH, United Kingdom

[5]Parent representative, United Kingdom

[6]Paediatric Intensive Care, Birmingham Children's Hospital, Birmingham Women's and Children's NHS Foundation Trust, Steelhouse Lane, Birmingham, B4 6NH, United Kingdom

[7]Paediatric Intensive Care Unit, Great Ormond Street Hospital for Children NHS Foundation Trust, and NIHR Biomedical Research Centre, WC1N 3JH, London, United Kingdom

[8]Respiratory Critical Care and Anaesthesia Unit, UCL Great Ormond Street Institute of Child Health, London, WC1N 1EH, United Kingdom

[9]School of Health & Society, University of Salford, Mary Seacole Building, Frederick Road Campus, Broad St, Frederick Road Campus, Salford, M6 6PU, United Kingdom

[10]Children’s Acute Transport Service, Great Ormond Street Hospital for Children NHS Foundation Trust and NIHR Biomedical Research Centre, WC1N 3JH, London, United Kingdom

[11]Paediatric Intensive Care Unit, St Mary's Hospital, Imperial College Healthcare NHS Trust, Praed Street, Paddington, London, W2 1NY, United Kingdom

For peer review only

Corresponding author

Dr Padmanabhan Ramnarayan

Consultant in Paediatric Intensive Care & Retrieval

Children's Acute Transport Service (CATS)

Heart and Lung Directorate

Great Ormond Street Hospital for Children NHS Foundation Trust

26-27 Boswell Street

London WC1N 3JZ

United Kingdom

Telephone: 020 7430 5850

Email: p.ramnarayan@gosh.nhs.uk

Keywords

non-invasive respiratory support, high flow nasal cannula, continuous positive airway pressure, randomised clinical trial

Word count: Abstract (296/300), Main manuscript (4000/4000)

For peer review only

For peer review only - http://bmjopen.bmj.com/site/about/guidelines.xhtml

Abstract

Introduction

Even though respiratory support is a common intervention in paediatric critical care, there is no randomised controlled trial (RCT) evidence regarding the effectiveness of two commonly used modes of non-invasive respiratory support (NRS), continuous positive airway pressure (CPAP) and high-flow nasal cannula therapy (HFNC). FIRST-ABC is a master protocol of two RCTs to evaluate the clinical and cost-effectiveness of HFNC (compared to CPAP) as the first-line mode of support in critically ill children.

Methods and analysis

We will recruit participants over a 30-month period at 25 UK paediatric critical care units (PICU/HDUs). Patients will be eligible if they are admitted/accepted for admission, aged >36 weeks corrected gestational age and <16 years, and assessed by the treating clinician to require non-invasive respiratory support for an acute illness (step-up RCT) or within 72 hours of extubation following a period of invasive ventilation (step-down RCT). Due to the emergency nature of the treatment, written informed consent will be deferred to after randomisation. Randomisation will occur 1:1 to CPAP or HFNC, stratified by site and age (<12 months vs. ≥12 months). The primary outcome is time to liberation from respiratory support for a continuous period of 48 hours. A total sample size of 600 patients in each RCT will provide 90% power with a type I error rate of 2.5% (one-sided) to exclude the pre-specified noninferiority margin of hazard ratio (HR) of 0.75. Primary analyses will be undertaken separately in each RCT in both the intention-to-treat and per-protocol populations.

Ethics and dissemination

This master protocol received favourable ethical opinion from NHS East of England - Cambridge South Research Ethics Committee (reference: 19/EE/0185) and approval from the Health Research Authority (reference: 260536). Results will be disseminated via publications in peer reviewed medical journals and presentations at national and international conferences.

Trial registration

ISRCTN60048867; Pre-results.

For peer review only - http://bmjopen.bmj.com/site/about/guidelines.xhtml

Article Summary

Strengths and limitations of this study

1. FIRST-ABC is a master protocol of the two largest RCTs to date to study the clinical and cost-effectiveness of high flow nasal cannula as the first-line mode of non-invasive respiratory support in critically ill children.

2. The FIRST-ABC master protocol includes two separate RCTs, one in acutely ill children requiring respiratory support (step-up RCT) and one in children requiring respiratory support after extubation from invasive ventilation (step-down RCT), to address the research question in two distinct but common clinical scenarios.

3. The design and conduct of FIRST-ABC has been informed by a successful pilot RCT that confirmed the feasibility of delivering a large pragmatic trial in critically ill children.

4. The choice of the primary outcome, time to liberation from all forms of respiratory support for a continuous period of at least 48 hours, was informed by clinicians as well as through patient and public involvement.

5. Changes to clinical practice during the trial period, and a resultant shift in equipoise regarding the choice of first-line mode of respiratory support in critically ill children, may affect the ability to recruit successfully to the RCTs.

For peer review only

For peer review only - http://bmjopen.bmj.com/site/about/guidelines.xhtml

Introduction

Nearly 75% of the 20,000 critically ill children admitted annually to United Kingdom (UK) paediatric intensive care units (PICUs) receive some form of respiratory support.[1] Increasing recognition of the risks of invasive ventilation has prompted greater use of non-invasive respiratory support (NRS) worldwide.[1 2] Two main modes of NRS are used, to support acutely ill children with respiratory failure or to provide post-extubation support after a spell of invasive ventilation.

Continuous positive airway pressure (CPAP) has been used by PICUs for over three decades.[3-5] Although observational data suggest that CPAP is effective, there have been few randomised clinical trials (RCTs) of CPAP in critically ill children.[5-7] CPAP can be uncomfortable and may be associated with complications such as air-leak and nasal trauma, often necessitating the use of sedation, close monitoring and a high level of nursing input. An alternate mode of NRS, high flow nasal cannula (HFNC), has gained popularity recently. It appears easy to use and is well-tolerated.[8-11] Between 16 and 35% of PICU admissions receive HFNC at some point during their stay.[1 12 13] The potential benefits of HFNC (improved patient comfort, safety profile and ease of nursing care), must be balanced against its potential risks (air leak, abdominal distension and nosocomial infection), and concerns regarding unnecessary prolongation of PICU/hospital stay and excess mortality from delayed escalation. There are few RCTs comparing HFNC with CPAP in the PICU setting. Previous RCTs do not include children with a range of ages and diagnoses needing either *step-up* or *step-down* (post-extubation) care, making it impossible to generalise their findings to contemporary practice.[14-16]

FIRST-ABC therefore addresses an important clinical dilemma faced daily by critical care clinicians: in a child requiring NRS, which modality, HFNC or CPAP, should they use as first-line therapy to achieve the best patient outcomes? Our research question was prioritised by clinicians as well as parents/patients. We previously successfully completed a pilot RCT, which supported the feasibility of performing a large pragmatic RCT comparing CPAP and HFNC in critically ill children, and informed its design and conduct.[17] This protocol has been written in accordance with the Standard Protocol Items: Recommendations for Interventional Trials (SPIRIT) statement.[18]

For peer review only - http://bmjopen.bmj.com/site/about/guidelines.xhtml

Methods

Aim

To evaluate the clinical and cost-effectiveness of the use of HFNC, as compared with CPAP, when used as the first-line mode in critically ill children requiring NRS:

A. for an acute illness (*step-up RCT*);
B. within 72 hours of extubation following a period of invasive ventilation (*step-down RCT*).

Primary objective

To evaluate the non-inferiority of HFNC, as compared with CPAP, when used as the first-line mode of NRS, both as a step-up treatment (*step-up RCT*) and as a step-down treatment (*step-down RCT*), on the time to liberation from respiratory support.

Design

FIRST-ABC is a master protocol comprising two pragmatic, multi-centre, parallel groups, non-inferiority RCTs (*step-up RCT* and *step-down RCT*) with shared infrastructure, including an internal pilot stage and integrated health economic evaluation. This design allows the research question to be addressed in each of the two important populations in an efficient way by minimising time and infrastructure costs as compared with conducting two sequential RCTs.[19]

A non-inferiority design was chosen based on previous RCTs in this area and feedback from clinicians indicating that the potential benefits of HFNC (in terms of patient comfort and ease of use) would mean that it would likely be preferred in usual practice even if not shown to be superior to CPAP.

Setting

FIRST-ABC is set in NHS paediatric critical care units (PICU and/or high dependency units (HDUs)) across England, Wales and Scotland. Sites are eligible to take part if they confirm collective equipoise regarding the choice of first-line NRS in their unit and commit to following trial procedures, including randomisation and data collection. Sites can start recruitment only after a site initiation visit and all relevant regulatory approvals.

Population

Critically ill children requiring NRS for (A) an acute illness (*step-up RCT*) or (B) within 72 hours of extubation following a period of invasive ventilation (*step-down RCT*).

For peer review only - http://bmjopen.bmj.com/site/about/guidelines.xhtml

Screening

Potentially eligible patients admitted/accepted for admission to the participating critical care unit will be screened against the inclusion/exclusion criteria by the local clinical/research team. For the step-up RCT, all admissions to the unit will be screened. For the step-down RCT, all patients extubated during unit admission will be screened. From these, Screening and Enrolment Logs will record enrolled patients, reasons for exclusion and reasons eligible patients are not enrolled.

Inclusion criteria

1) Admitted/Accepted for admission to PICU/HDU
2) Age >36 weeks corrected gestational age and <16 years
3) Assessed by the treating clinician to require non-invasive respiratory support, EITHER
   A. for an acute illness (*step-up RCT*) OR
   B. within 72 hours of extubation following a period of invasive ventilation (*step-down RCT*).

Exclusion criteria

1) Assessed by the treating clinician to require immediate intubation and invasive ventilation due to severe hypoxia, acidosis and/or respiratory distress, upper airway obstruction, inability to manage airway secretions or recurrent apnoeas
2) Tracheostomy in place
3) Received HFNC/CPAP for >2 hours in the prior 24 hours
4) On home non-invasive ventilation prior to PICU/HDU admission
5) Presence of untreated air-leak (pneumothorax/pneumomediastinum)
6) Midfacial/craniofacial anomalies (unrepaired cleft palate, choanal atresia) or recent craniofacial surgery
7) Agreed 'not for intubation' or other limitation of critical care treatment plan in place.
8) Previously recruited to FIRST-ABC (step-up RCT or step-down RCT on this or a previous admission)
9) Clinician decision to start other form of non-invasive respiratory support (i.e. not HFNC or CPAP)

Randomisation

Randomisation will be performed after confirming eligibility and as soon as possible to the anticipated start of the randomised treatment. In each RCT, eligible patients will be randomised in a 1:1 ratio to either CPAP or HFNC using a central telephone/web-based randomisation service available 24 hours/7 days a week. The randomisation sequence will be computer

For peer review only - http://bmjopen.bmj.com/site/about/guidelines.xhtml

generated and variable block sizes will be used to strengthen allocation concealment. Randomisation will be stratified by site and age (<12 months versus ≥12 months).

The randomised treatment will be commenced as soon as practically possible. Following randomisation, each participant will be assigned a unique FIRST-ABC Trial Number and a Case Report Form (CRF) completed by the local research team.

Delivery of HFNC

Any approved medical device capable of delivering heated, humidified, high flow through nasal cannulae can be used to provide HFNC at the prescribed gas flow rates during the trial period. To standardise treatment, clinical criteria and guidance for the initiation, maintenance and weaning of HFNC are provided in a trial algorithm (Figure 1). The trial recommends that patients are assessed for response to the treatment, readiness to wean and for stopping HFNC, as per the HFNC algorithm, at least twice per day (e.g. at ward rounds).

For peer review only

For peer review only - http://bmjopen.bmj.com/site/about/guidelines.xhtml

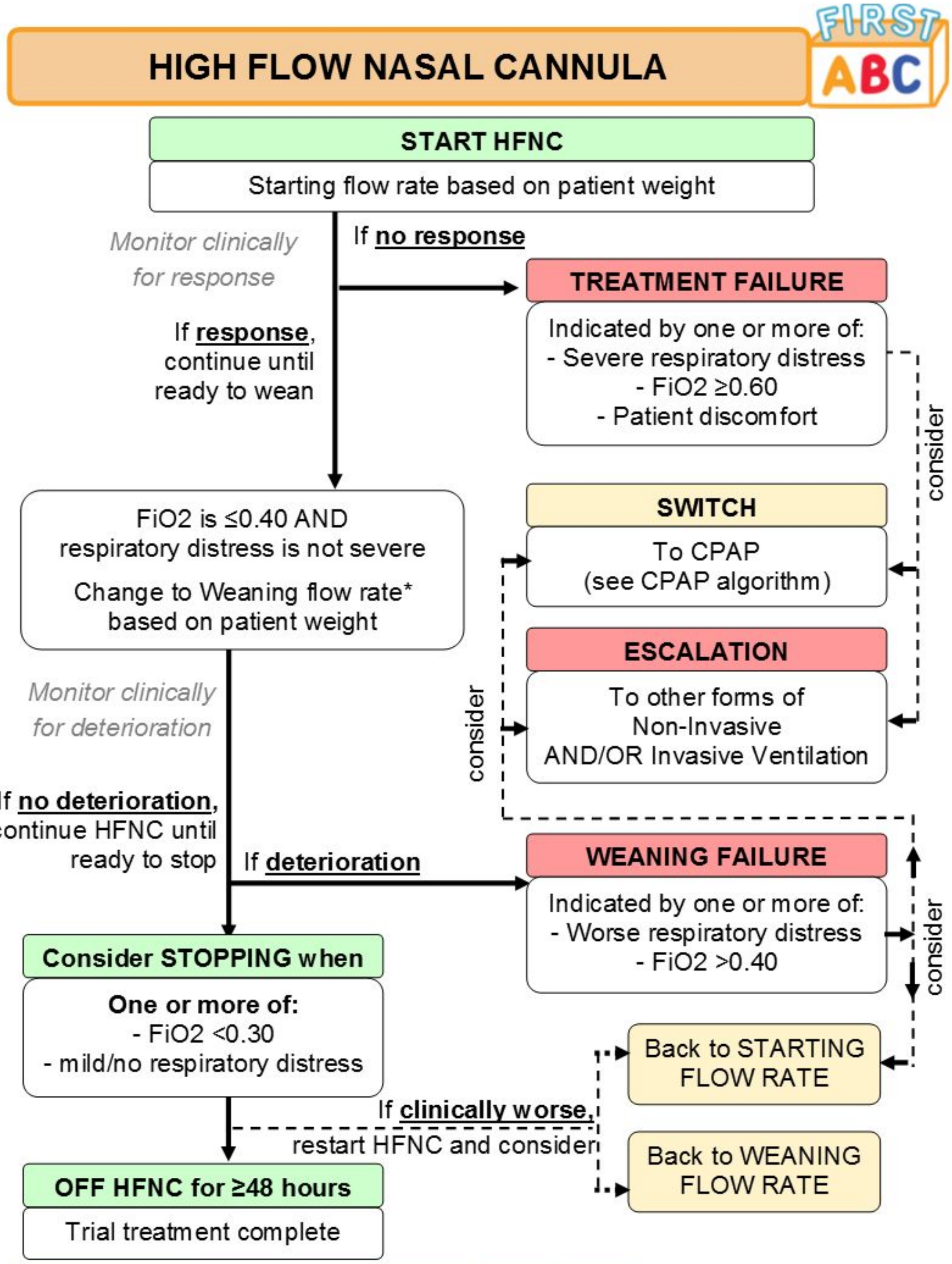

Weight (kg)	≤12	13-15	16-30	31-50	>50
Starting flow rate	2 l/min/kg	25-30 l/min	35 l/min	40 l/min	50 l/min
Weaning flow rate	1 l/min/kg	13-15 l/min	18 l/min	20 l/min	25 l/min

Figure 1. Algorithm for delivery of High Flow Nasal Cannula.

For peer review only - http://bmjopen.bmj.com/site/about/guidelines.xhtml

Delivery of CPAP

CPAP will be started using an approved medical device at a set expiratory pressure of 7-8 cm H_2O. The trial does not specify any particular device or patient interface for the provision of CPAP. To standardise treatment, clinical criteria and guidance for the initiation, maintenance and weaning of CPAP are provided in a trial algorithm (Figure 2). It is recommended that patients are assessed for response to the treatment, readiness to wean and for stopping CPAP, as per the CPAP algorithm, at least twice per day (e.g. at ward rounds).

Clinical practice during the trial

Since staff in participating sites already use HFNC and CPAP, no additional training related to the use of HFNC or CPAP will be provided for the trial, but resources for training in the trial algorithms will be provided. As the medical devices and interfaces that deliver HFNC and CPAP are easily distinguishable from each other, it will not be possible to blind the patient, parents/guardians or clinical staff.

The trial algorithms will be followed until the patient has been liberated from all forms of respiratory support for at least 48 continuous hours. As per current practice, clinicians will be able to stop HFNC/CPAP and switch to the other treatment or escalate to other forms of respiratory support, if clinically deemed necessary. Pre-specified objective criteria to identify non-responders to HFNC/CPAP are provided in the algorithms as a guide for clinicians considering switching or escalating respiratory support. Reasons for switches or escalations will be recorded. Patients who switch or escalate treatments will remain in the trial and continue to be monitored until liberation from respiratory support. All other usual care will be at the discretion of the treating clinical team

For peer review only

For peer review only - http://bmjopen.bmj.com/site/about/guidelines.xhtml

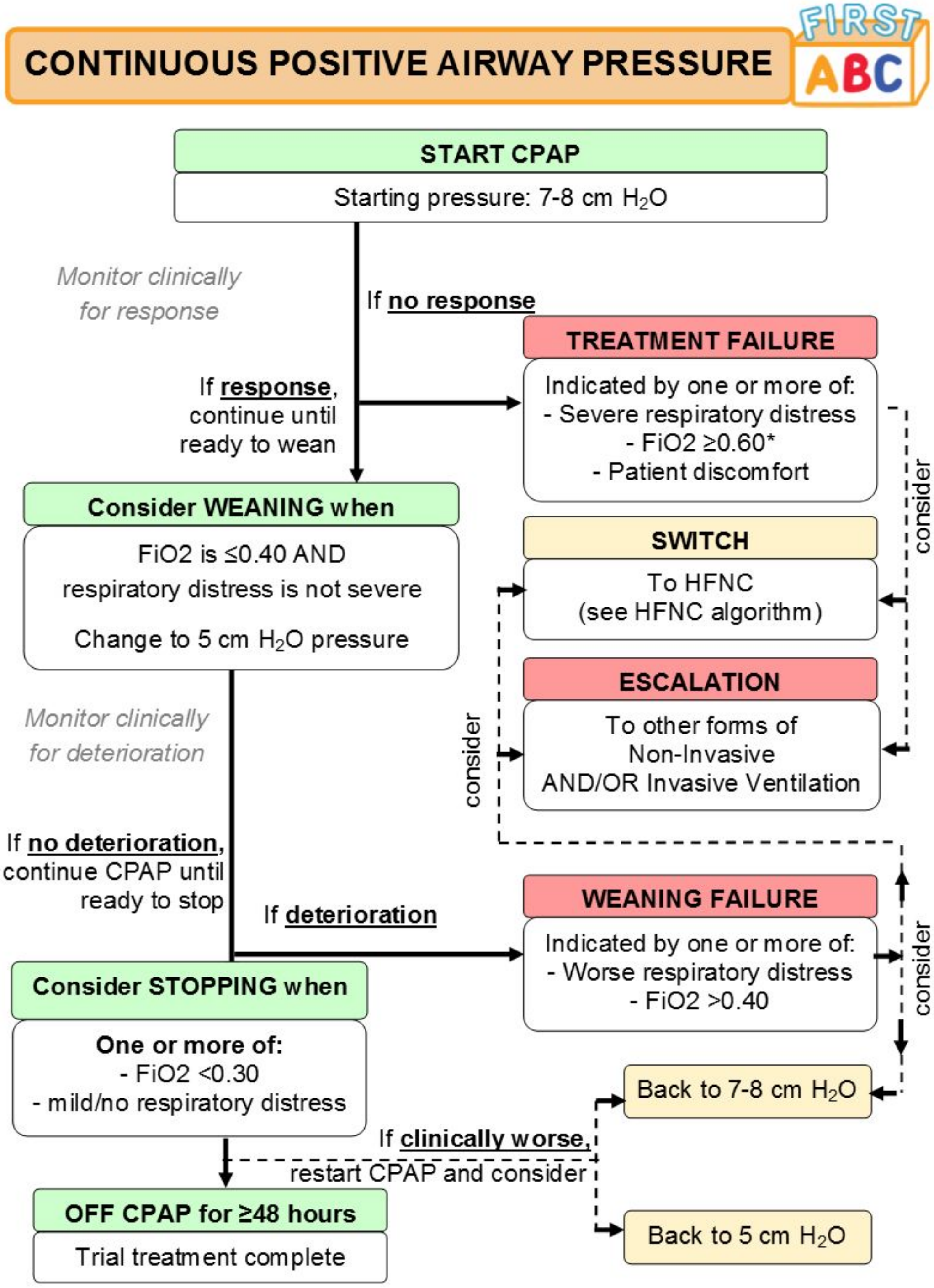

Figure 2. Algorithm for delivery of Continuous Positive Airway Pressure.

For peer review only - http://bmjopen.bmj.com/site/about/guidelines.xhtml

Consent procedures

Consent will be sought for the child (patient) from their parent/legal guardian. Children become eligible for FIRST-ABC when critically ill, a profoundly stressful time for parents/guardians, during which there are ethical concerns both about the burden of trying to understand the trial and the ability to provide informed consent. Initiation of NRS typically occurs during a time-sensitive situation, where delays could be detrimental to the child and to the trial's scientific validity. Moreover, both CPAP and HFNC are already widely used in standard practice across the NHS. Considering these reasons, FIRST-ABC has been given ethical approval to use a deferred consent model ('research without prior consent'). Once a patient is confirmed eligible, they will be randomised and the allocated treatment (CPAP or HFNC) commenced as soon as possible. This model, developed in line with the CONseNt methods in paediatric Emergency and urgent Care Trials (CONNECT) guidance,[20] has been found acceptable to parents/guardians and clinicians in several recent RCTs in the PICU setting[17 21-25] and is informed by experience/feedback from the pilot RCT.[17]

Following randomisation, a trained, delegated member of the local research team will approach the child's parents/guardians as soon as appropriate and practically possible to discuss the trial (usually within 24-48 hours of randomisation). A Participant Information Sheet (PIS) will be provided, covering information about the purpose of the trial; the consequences of participating or not; confidentiality; use of personal data; data security; and the future availability of the trial results. A Consent Form will be provided, indicating that: the information given has been read and understood; participation is voluntary and consent can be withdrawn at any time without consequence; and that consent is given for access to medical records to continue data collection, to receive a follow-up questionnaire and for anonymised data to be shared in the future. Due to age and severity of illness, it will not be possible to involve the patient in the consenting process. Instead, assent will be obtained prior to hospital discharge if their condition allows (e.g. they regain mental capacity).

A modification of the consent procedure will be utilised for two rare situations where either the patient: a) is discharged from hospital prior to obtaining consent, or b) dies prior to consent being sought. In the former, the local research team will follow up with the parent/guardian, initially by phone and then by post, for consent. Postal contact will be made again if there is no response after four weeks. If no Consent Form is received within four weeks of the second letter, the participant will be included in the trial unless they notify the research team otherwise. In the latter

For peer review only - http://bmjopen.bmj.com/site/about/guidelines.xhtml

situation, the local research team will obtain information from colleagues and bereavement counsellors to establish the most appropriate clinical/research team member to notify the parents/guardians of involvement in the trial. If approach for consent is deemed not appropriate prior to the parent/guardian's departure from hospital, then they will be approached by post four weeks post-randomisation. The letter will explain how to opt out of the trial. Postal contact will be made again if there is no response after four weeks. If no Consent Form is received within four weeks of the second letter, the participant's data will be included in the trial.

If informed consent is refused or withdrawn, this decision will be respected and abided by, and no further contact made. All data occurring up to the point of this decision will be retained in the trial, unless parents/guardians request otherwise.

Safety monitoring

Adverse Event (AE) reporting will follow the Health Research Authority guidelines on safety reporting in studies which do not use Investigational Medicinal Products (non-CTIMPs). The following events have been pre-specified as potential AEs that could be related to CPAP and/or HFNC and observed in participants from the date and time of randomisation until 48 hours of liberation from all forms of respiratory support:

1. Nasal trauma
2. Facial/neck trauma
3. Abdominal distension
4. Pneumothorax
5. Pneumomediastinum
6. Subcutaneous emphysema
7. Facial thermal injury
8. Respiratory arrest
9. Cardiac arrest
10. Aspiration

Occurrences of the specified, expected adverse events will be recorded for all randomised patients. Considering that eligible patients are critically ill and at increased risk of experiencing AEs, occurrences of non-specified, adverse events will only be reported if considered to be related to either CPAP or HFNC (i.e. 'possibly', 'probably' or 'definitely' – see Supplement 1 for definitions). Any event classified as 'severe' or 'life-threatening' in severity is considered a

For peer review only - http://bmjopen.bmj.com/site/about/guidelines.xhtml

For peer review only

Serious Adverse Event (SAE) and must be reported to ICNARC CTU. If the SAE is evaluated by the Trial Management Group (TMG) as a related and unexpected SAE, the ICNARC CTU will submit a report to the REC within 15 calendar days.

Questionnaire follow-up

At six months, after assessing the child's survival status, each consenting parent will be sent a questionnaire (via email or post) by the ICNARC CTU to assess Health-related Quality of Life (HrQoL) and health service/resource use. Non-responders will be followed-up by telephone three weeks later.

For peer review only

Outcome measures

Primary outcome

- Time to liberation from respiratory support, defined as the start of a 48-hour period during which the child was free of all forms of respiratory support (not including supplemental oxygen alone).

Secondary outcomes

- Mortality at PICU/HDU discharge, day 60 and day 180
- Rate of (re)intubation at 48 hours
- Duration of PICU/HDU and hospital stay
- Patient comfort, during randomised treatment and during non-invasive respiratory support (i.e. HFNC and/or CPAP), assessed using the COMFORT-B score[26]
- Proportion of patients in whom sedation is used during non-invasive respiratory support
- Parental stress, in hospital at/around the time of consent at 24-48 hours, measured using the Parental Stressor Scale: PICU[27]
- HrQoL at six months using age-appropriate Pediatric Quality of Life Inventory (Peds-QL)[32] and Child Health Utility (CHU-9D) questionnaire[28]

Cost effectiveness analysis (CEA) outcomes

- Total costs at six months
- Quality-Adjusted Life-Years (QALYs) at six months
- Incremental net monetary benefit gained at a willingness-to-pay of £20,000 per QALY at six months associated with HFNC versus CPAP[29]

For peer review only - http://bmjopen.bmj.com/site/about/guidelines.xhtml

Data collection

To maximise efficiency, FIRST-ABC collaborates with the Paediatric Intensive Care Audit Network (PICANet) to make best use of established PICU data collection infrastructure. Where possible, recruited patients will be consented for data linkage with routine sources (e.g. national death registration data via NHS Digital or equivalent). Additional trial-specific data collection items are limited to the minimum required to deliver trial objectives (**Error! Reference source not found.**).

Table 1 Patient data collection schedule

For peer review only

	Baseline	At time of consent	During non-invasive respiratory support	End of PICU/HDU stay	End of hospital stay	At six months
**In-hospital**						
Clinical/baseline data	✓					
Patient/parent details		✓				
Types of respiratory support received*	✓		✓			
Patient comfort and sedation use			✓			
Parental stress		✓				
Discharge data				✓	✓	
Safety monitoring data			✓			
**At follow-up**						
PedsQL						✓
CHU-9D						✓
Health services/ resource use						✓

PICU: paediatric intensive care unit, HDU: high dependency unit, PedsQL: Pediatric Quality of Life Inventory (Peds-QL), CHU-9D: Child Health Utility questionnaire.

**including weaning, switches and escalations from High Flow Nasal Cannula (HFNC)/Continuous Positive Airway Pressure (CPAP)*

For peer review only - http://bmjopen.bmj.com/site/about/guidelines.xhtml

All participant data will be entered onto the secure electronic case report form and undergo validation checks for completeness, accuracy and consistency. The site Principal Investigator will oversee and be responsible for data collection, quality and recording.

Statistical Methods

Sample size

To achieve 90% power with a type I error rate of 2.5% (one-sided) to exclude the pre-specified noninferiority margin of HR=0.75 requires 508 events to be observed. Based on pilot RCT data,[17] we anticipate 5% censoring due to death or transfer, leading to a required sample size of 268 patients per group in each of the two RCTs. To allow for withdrawal/refusal of consent, and for exclusion due to non-adherence in the per-protocol (PP) population, we will recruit a total sample size of 600 patients in each RCT.

Internal pilot

Data will be analysed at the end of the internal pilot stage (months 7-12 of the grant timeline) on patients recruited during the first six months in each RCT. The RCTs will progress from pilot to full trial based on pre-specified progression criteria related to successful site set-up, screening and recruitment, and adherence. The final decision on progression will be made by the funder after recommendation, or not, by the Trial Steering Committee (TSC).

Clinical effectiveness analysis

All analyses will be publicly lodged in a statistical analysis plan, a priori, before the investigators are unblinded to any trial outcomes. Following best practice for non-inferiority trials, the primary analyses will be undertaken in both intention-to-treat (ITT) and PP populations, with robust conclusions possible in the situation where both populations provide concordant results. Results will be reported in accordance with the CONSORT statement extension for non-inferiority and equivalence trials.[30]

Analyses will be undertaken independently for each RCT. In each RCT, baseline patient characteristics will be compared between the two groups to observe balance and the success of randomisation. These comparisons will not be subjected to statistical testing. The delivery of the intervention will be described for each group in detail, including number and percentage of patients who commence the randomised treatment, remain on the randomised treatment until liberation from ventilation, who are changed to a different mode of respiratory support.

For peer review only - http://bmjopen.bmj.com/site/about/guidelines.xhtml

HFNC will be considered non-inferior to CPAP if the lower bounds of the 95% confidence intervals for the hazard ratio (HR) from Cox regression models on time to liberation from respiratory support fitted in both the ITT and PP populations exclude the pre-specified non-inferiority margin of 0.75 (corresponding to approximately a 16-hour increase in median time to liberation, based on pilot RCT data). This margin was considered adequate such that the other potential benefits of HFNC in terms of comfort and tolerability would mean that it would be likely to be preferred in usual practice. The Cox regression models will be adjusted for important baseline characteristics. The covariates for inclusion in the regression models will be selected a priori based on an established relationship with outcome for critically ill children, and not because of observed imbalance, significance in univariable analyses or by a stepwise selection method.

Subgroup analyses for the primary outcome will be performed to test for interactions between the effect of allocated treatment group and the following baseline covariates:

- age (<12 months versus ≥12 months)
- severity of respiratory distress at randomisation (severe versus mild/moderate)
- Co-morbidities (None versus Neurological/neuromuscular versus Other)
- Sp02/Fi02 (SF) ratio at randomisation
- for step-up RCT only:
  - clinical indication (bronchiolitis versus other respiratory (airway problem, asthma/wheeze or any other respiratory) versus cardiac versus other (neurological, sepsis/infection, any other))
  - whether child was on NRS at randomisation (Yes/No)
- for step-down RCT only:
  - length of prior invasive mechanical ventilation (<5 days versus ≥5 days)
  - reason for invasive mechanical ventilation (cardiac versus other)
  - planned (randomisation followed by extubation) vs rescue (extubation followed by randomisation) non-invasive respiratory support

As a sensitivity analysis, the primary analysis will be repeated using time to start weaning of NRS (i.e. duration of 'acute' respiratory support) and time to meeting objective 'readiness to wean NRS' criteria.

For peer review only - http://bmjopen.bmj.com/site/about/guidelines.xhtml

Secondary analyses of binary outcomes (mortality, reintubation) will be performed by Fisher's exact test and adjusted logistic regression. Duration of survival to day 180 will be plotted as Kaplan-Meier survival curves, compared unadjusted with the log rank test and adjusted using Cox regression models. Analyses of duration of PICU/HDU and hospital stay will be performed by Wilcoxon rank-sum tests, stratified by survival status. Analyses of COMFORT-B score, sedation use, PSS:PICU and HrQoL will be performed by t-tests and adjusted linear regression.

In the step-up RCT, a single interim analysis will be carried out after the recruitment and follow-up to day 60 of 300 patients. The interim analysis will use a Peto-Haybittle stopping rule to recommend early termination due to superiority of either intervention ($P<0.001$) in time to liberation from respiratory support or evidence of harm from either intervention ($P<0.05$) in mortality at day 60. Both tests will be performed using a log-rank test on all available data within the ITT population. Further interim analyses will be performed only if requested by the Data Monitoring and Ethics Committee (DMEC).

In the step-down RCT, due to faster than anticipated recruitment, no formal interim analysis will be performed. Safety data (counts and percentages of adverse events by arm, and a line listing of SAEs) will be available for scrutiny by the DMEC, by the end of the internal pilot stage.

Integrated health economic evaluation

The CEA will take an NHS and Personal Social Services perspective.[29] Patient-level resource use data will be obtained from CRFs, PICANet, and a health services questionnaire (HSQ). Resource use data from the PICU/HDU stay will be taken from the CRF and linked routine data from PICANet. Information on subsequent PICU/HDU and hospital admissions will be obtained via data linkage with PICANet and through completion of the HSQ. Data on the level of care for PICU bed-days will be gathered through routine collection of the Paediatric Critical Care Minimum Dataset (PCCMDS) in the participating sites via the PICANet database. Use of primary care and community health services will be assessed by HSQ at six months. Patient-level resource use data will be combined with appropriate unit costs from the NHS payment by results and Personal Social Services Research Unit databases to report total costs per patient for up to six months post-randomisation. Data from PedsQL and CHU-9D at six months will be combined with survival data to report QALYs at six months. The CEA will follow the intention-to-treat principle and report the mean (95% confidence interval) incremental costs, QALYs and net monetary benefit at six months. The CEA will use multilevel linear regression models that allow

For peer review only - http://bmjopen.bmj.com/site/about/guidelines.xhtml

for clustering of patients at site. The analysis will adjust for key baseline covariates at both patient and site level.

Governance and oversight

Research ethics

The trial received favourable ethical opinion from NHS East of England - Cambridge South Research Ethics Committee (reference number: 19/EE/0185) and approval from the Health Research Authority (Integrated Research Application System (IRAS) number: 260536). Evidence of local confirmation of capacity and capability at each site must be provided to the ICNARC CTU prior to site activation.

Confidentiality

ICNARC CTU will act to preserve participant confidentiality and will not disclose or reproduce any information by which participants could be identified. All data will be stored securely.

Oversight

The TMG, led by the Chief Investigator, is responsible for the management of FIRST-ABC. It meets regularly and includes the Investigators and ICNARC CTU trial team. FIRST-ABC is managed by the ICNARC CTU in accordance with the Medical Research Council's Good Research Practice: Principles and Guidelines[31] which is based on the International Conference on Harmonisation guidelines on Good Clinical Practice[32] principles and the UK Department of Health's Policy Framework for Health and Social Care Research.[33] The on-site monitoring plan will follow a risk-based strategy.

A majority independent TSC has been established to monitor trial progress and includes PPI representatives, experienced clinicians and researchers, in addition to the Chief Investigator and Head of Research at ICNARC. An independent DMEC has been established to monitor patient recruitment and retention, adherence and safety.

The Great Ormond Street Hospital for Children NHS Foundation Trust are the trial sponsor (reference: 17IA05). As the sponsor is an NHS organisation, NHS indemnity will apply for legal liability arising from the design, management and conduct of the research.

For peer review only - http://bmjopen.bmj.com/site/about/guidelines.xhtml

Patient and Public Involvement (PPI)

We had considerable PPI input into the pilot RCT[17] as well as the main trial described here. The parent of a child who received respiratory support is a co-investigator and has actively contributed to the trial design and procedures, including the use of deferred consent.

Trial status

This paper presents the master protocol (v1.2, dated 23 January 2020)[34] for the two largest RCTs studying the clinical and cost effectiveness of HFNC therapy as the first-line mode of NRS in critically ill children. It will provide robust evidence for the two distinct but common clinical scenarios in which NRS is primarily used . The first participant was recruited in August 2019. At the time of submission, patient recruitment was ongoing – with recruitment planned to complete in November 2020 and January 2022 for the step-down RCT and step-up RCT, respectively. Each RCT will be disseminated independently, including through publication in peer-reviewed medical journals and at national and international conferences.

For peer review only

For peer review only - http://bmjopen.bmj.com/site/about/guidelines.xhtml

References

1. Paediatric Intensive Care Audit Network (PICANet). Annual Report 2019. Leeds: Universities of Leeds and Leicester, 2019.
2. Wolfler A, Calderini E, Iannella E, et al. Evolution of Noninvasive Mechanical Ventilation Use: A Cohort Study Among Italian PICUs. Pediatric critical care medicine : a journal of the Society of Critical Care Medicine and the World Federation of Pediatric Intensive and Critical Care Societies 2015;**16**(5):418-27.
3. Shah PS, Ohlsson A, Shah JP. Continuous negative extrathoracic pressure or continuous positive airway pressure compared to conventional ventilation for acute hypoxaemic respiratory failure in children. The Cochrane database of systematic reviews 2013(11):CD003699.
4. Jat KR, Mathew JL. Continuous positive airway pressure (CPAP) for acute bronchiolitis in children. The Cochrane database of systematic reviews 2015;**1**:CD010473.
5. Donlan M, Fontela PS, Puligandla PS. Use of continuous positive airway pressure (CPAP) in acute viral bronchiolitis: a systematic review. Pediatric pulmonology 2011;**46**(8):736-46.
6. Thia LP, McKenzie SA, Blyth TP, et al. Randomised controlled trial of nasal continuous positive airways pressure (CPAP) in bronchiolitis. Archives of disease in childhood 2008;**93**(1):45-7.
7. Peters MJ, Agbeko R, Davis P, et al. Randomized Study of Early Continuous Positive Airways Pressure in Acute Respiratory Failure in Children With Impaired Immunity (SCARF) ISRCTN82853500. Pediatric critical care medicine : a journal of the Society of Critical Care Medicine and the World Federation of Pediatric Intensive and Critical Care Societies 2018;**19**(10):939-48.
8. Lee JH, Rehder KJ, Williford L, et al. Use of high flow nasal cannula in critically ill infants, children, and adults: a critical review of the literature. Intensive care medicine 2013;**39**(2):247-57.
9. Spentzas T, Minarik M, Patters AB, et al. Children with respiratory distress treated with high-flow nasal cannula. Journal of intensive care medicine 2009;**24**(5):323-8.
10. Ramnarayan P, Schibler A. Glass half empty or half full? The story of high-flow nasal cannula therapy in critically ill children. Intensive care medicine 2017;**43**(2):246-49.
11. Morris JV, Kapetanstrataki M, Parslow RC, et al. Patterns of Use of Heated Humidified High-Flow Nasal Cannula Therapy in PICUs in the United Kingdom and Republic of Ireland*. Pediatric Critical Care Medicine 2019;**20**(3):223-32.
12. Coletti KD, Bagdure DN, Walker LK, et al. High-Flow Nasal Cannula Utilization in Pediatric Critical Care. Respir Care 2017;**62**(8):1023-29.
13. Baudin F, Gagnon S, Crulli B, et al. Modalities and Complications Associated With the Use of High-Flow Nasal Cannula: Experience in a Pediatric ICU. Respir Care 2016;**61**(10):1305-10.
14. Mayfield S, Jauncey-Cooke J, Hough JL, et al. High-flow nasal cannula therapy for respiratory support in children. The Cochrane database of systematic reviews 2014(3):Cd009850.
15. Beggs S, Wong ZH, Kaul S, et al. High-flow nasal cannula therapy for infants with bronchiolitis. The Cochrane database of systematic reviews 2014;**1**:CD009609.
16. Milesi C, Essouri S, Pouyau R, et al. High flow nasal cannula (HFNC) versus nasal continuous positive airway pressure (nCPAP) for the initial respiratory management of acute viral bronchiolitis in young infants: a multicenter randomized controlled trial (TRAMONTANE study). Intensive care medicine 2017;**43**(2):209-16.
17. Ramnarayan P, Lister P, Dominguez T, et al. FIRST-line support for Assistance in Breathing in Children (FIRST-ABC): a multicentre pilot randomised controlled trial of high-flow nasal cannula therapy versus continuous positive airway pressure in paediatric critical care. Crit Care 2018;**22**(1):144.

For peer review only - http://bmjopen.bmj.com/site/about/guidelines.xhtml

18. Chan AW, Tetzlaff JM, Altman DG, et al. SPIRIT 2013 statement: defining standard protocol items for clinical trials. Ann Intern Med 2013;**158**(3):200-7.
19. Woodcock J, LaVange LM. Master Protocols to Study Multiple Therapies, Multiple Diseases, or Both. N Engl J Med 2017;**377**(1):62-70.
20. CONseNt methods in paediatric Emergency and urgent Care Trials. Secondary CONseNt methods in paediatric Emergency and urgent Care Trials. https://www.liverpool.ac.uk/psychology-health-and-society/research/connect/.
21. Harron K, Woolfall K, Dwan K, et al. Deferred Consent for Randomized Controlled Trials in Emergency Care Settings. Pediatrics 2015;**136**(5):e1316-22.
22. Woolfall K, Frith L, Gamble C, et al. How parents and practitioners experience research without prior consent (deferred consent) for emergency research involving children with life threatening conditions: a mixed method study. BMJ Open 2015;**5**(9):e008522.
23. Woolfall K, Frith L, Gamble C, et al. How experience makes a difference: practitioners' views on the use of deferred consent in paediatric and neonatal emergency care trials. BMC Med Ethics 2013;**14**:45.
24. O'Hara CB, Canter RR, Mouncey PR, et al. A qualitative feasibility study to inform a randomised controlled trial of fluid bolus therapy in septic shock. Archives of disease in childhood 2018;**103**(1):28-32.
25. Peters MJ, Jones GAL, Wiley D, et al. Conservative versus liberal oxygenation targets in critically ill children: the randomised multiple-centre pilot Oxy-PICU trial. Intensive care medicine 2018;**44**(8):1240-48.
26. van Dijk M, Peters JWB, van Deventer P, et al. The COMFORT Behavior Scale: a tool for assessing pain and sedation in infants. Am J Nurs 2005;**105**(1):33-36.
27. Carter MC, Miles MS. The Parental Stressor Scale: Pediatric Intensive Care Unit. Maternal-child nursing journal 1989;**18**(3):187-98.
28. Stevens K. Valuation of the Child Health Utility 9D Index. PharmacoEconomics 2012;**30**(8):729-47.
29. National Institute for Health and Care Excellence (NICE). Guide to the methods of technology appraisal, 2013.
30. Piaggio G, Elbourne DR, Pocock SJ, et al. Reporting of noninferiority and equivalence randomized trials: extension of the CONSORT 2010 statement. JAMA 2012;**308**(24):2594-604.
31. Medical Research Council. Good Research Practice: Principles and Guidelines Secondary Good Research Practice: Principles and Guidelines 2012. https://mrc.ukri.org/publications/browse/good-research-practice-principles-and-guidelines/.
32. INTERNATIONAL CONFERENCE ON HARMONISATION OF TECHNICAL REQUIREMENTS FOR REGISTRATION OF PHARMACEUTICALS FOR HUMAN USE. GUIDELINE FOR GOOD CLINICAL PRACTICE. 1996
33. Health Research Authority. UK policy framework for health and social care research. 2017.
34. National Institute for Health Research. FIRST-line support for Assistance in Breathing in Children (FIRST-ABC): a master protocol of two randomised trials to evaluate the non-inferiority of high flow nasal cannula therapy versus continuous positive airway pressure for non-invasive respiratory support in paediatric critical care. Secondary FIRST-line support for Assistance in Breathing in Children (FIRST-ABC): a master protocol of two randomised trials to evaluate the non-inferiority of high flow nasal cannula therapy versus continuous positive airway pressure for non-invasive respiratory support in paediatric critical care 2019. https://www.journalslibrary.nihr.ac.uk/programmes/hta/179428/#/.

For peer review only - http://bmjopen.bmj.com/site/about/guidelines.xhtml

Footnotes

Acknowledgements

The authors thank Karen Thomas, Izabella Orzechowska, Michelle Saull and Roger Parslow for their contribution to the set-up and delivery of FIRST-ABC. The authors also thank the research and clinical staff at the participating sites: Addenbrookes' Hospital (Cambridge), Alder Hey Children's Hospital (Liverpool), Birmingham Women and Children's Hospital, Bristol Royal Hospital for Children, Chelsea and Westminster Hospital, Evelina London Children's Hospital, Great North Children's Hospital (Newcastle), Great Ormond Street Hospital (London), Hull Royal Infirmary, James Cook University Hospital (Middlesbrough), John Radcliffe Hospital (Oxford), King's College Hospital (London), Leicester Royal Infirmary and Glenfield Hospital, Noah's Ark Children's Hospital for Wales (Cardiff), Queens Medical Centre (Nottingham), Royal Alexandra Children's Hospital (Brighton), Royal Brompton Hospital (London), Royal Hospital for Sick Children Edinburgh, Royal Manchester Children's Hospital, Sheffield Children's Hospital, Southampton Children's Hospital, St George's Hospital (London), St Mary's Hospital (London) and The Royal London Hospital. The authors acknowledge the UK Paediatric Intensive Care Society – Study Group for supporting the trial.

Contributors

PR is Chief Investigator. ARB is Trial Manager. PR and ARB drafted the manuscript.
PD, RF, RG, DH, JL, KM, PM, RP, MP, KR, SZ, and LT are trial co-applicants and members of the Trial Management Group. LD supported management of the trial. All authors read and approved the final version.

Funding

This trial is funded by the National Institute for Health Research (NIHR) Health Technology Assessment (HTA) Programme (project number: 17/94/28). Great Ormond Street Hospital for Children NHS Foundation Trust is the trial sponsor. The views expressed are those of the author(s) and not necessarily those of the NIHR or the Department of Health and Social Care nor the Sponsor. The funder and sponsor had no role in the writing of this article or the decision to submit the protocol for publication.

Competing interests

None declared.

For peer review only

For peer review only - http://bmjopen.bmj.com/site/about/guidelines.xhtml

Patient consent

Not required.

Ethics approval

The trial has received a favourable ethical opinion from the NHS East of England - Cambridge South Research Ethics Committee (reference number: 19/EE/0185) and approval from the Health Research Authority (Integrated Research Application System (IRAS) number: 260536).

For peer review only

For peer review only - http://bmjopen.bmj.com/site/about/guidelines.xhtml

Supplement 1 – Safety monitoring definitions

Severity

- **None**: indicates no event or complication.

- **Mild**: complication results in only temporary harm and does not require clinical treatment.

- **Moderate**: complication requires clinical treatment but does not result in significant prolongation of hospital stay. Does not usually result in permanent harm and where this does occur the harm does not cause functional limitation to the participant.

- **Severe**: complication requires clinical treatment and results in significant prolongation of hospital stay, permanent functional limitation.

- **Life-threatening:** complication that may lead to death or where the participant died as a direct result of the complication/adverse event.

Relatedness

- **None**: there is no evidence of any relationship to the study treatment.

- **Unlikely:** There is little evidence to suggest a causal relationship (e.g. because the event did not occur within a reasonable time after administration of the trial treatment). There is another reasonable explanation of the event (e.g. the participant's clinical condition, other concomitant medications).

- **Possibly:** There is some evidence to suggest a causal relationship (e.g. because the event occurs within a reasonable time after administration of the trial procedure). However, the influence of other factors may have contributed to the event (e.g. the participant's clinical condition, other concomitant medications).

For peer review only

For peer review only - http://bmjopen.bmj.com/site/about/guidelines.xhtml

- **Probably:** There is evidence to suggest a causal relationship and the influence of other factors is unlikely.

- **Definitely:** There is clear evidence to suggest a causal relationship and other possible contributing factors can be ruled out.

Expectedness

- **Expected**: the event is listed as an expected AE (see ***Error! Reference source not found.***).

- **Unexpected**: the event is not listed as an expected AE (see ***Error! Reference source not found.***).

For peer review only

For peer review only - http://bmjopen.bmj.com/site/about/guidelines.xhtml

SPIRIT

STANDARD PROTOCOL ITEMS: RECOMMENDATIONS FOR INTERVENTIONAL TRIALS

SPIRIT 2013 Checklist: Recommended items to address in a clinical trial protocol and related documents*

Section/item	Item No	Description	Page No.
**Administrative information**			
Title	1	Descriptive title identifying the study design, population, interventions, and, if applicable, trial	P1
Trial registration	2a	Trial identifier and registry name. If not yet registered, name of intended registry	P3
	2b	All items from the World Health Organization Trial Registration Data Set	Throughout
Protocol version	3	Date and version identifier	P20
Funding	4	Sources and types of financial, material, and other support	P24
Roles and responsibilities	5a	Names, affiliations, and roles of protocol contributors	P24
	5b	Name and contact information for the trial sponsor	P19
	5c	Role of study sponsor and funders, if any, in study design; collection, management, analysis, and interpretation of data; writing of the report; and the decision to submit the report for publication, including whether they will have ultimate authority over any of [illegible]	P24
	5d	Composition, roles, and responsibilities of the coordinating centre, steering committee, endpoint adjudication committee, data management team, and other individuals or groups overseeing the trial, if applicable (see Item 21a for data monitoring committee)	P19
**Introduction**			
Background and rationale	6a	Description of research question and justification for undertaking the trial, including summary of relevant studies (published and unpublished) examining benefits and harms for each intervention	P4
	6b	Explanation for choice of comparators	P4
Objectives	7	Specific objectives or hypotheses	P5

For peer review only

Trial design	8	Description of trial design including type of trial (eg, parallel group, crossover, factorial, single group), allocation ratio, and framework (eg, superiority,	P5

For peer review only

Methods: Participants, interventions, and outcomes

Study setting	9	Description of study settings (eg, community clinic, academic hospital) and list of countries where data will be collected. Reference to where list of study sites can be obtained	P5
Eligibility criteria	10	Inclusion and exclusion criteria for participants. If applicable, eligibility criteria for study centres and individuals who will perform the interventions (eg, surgeons, psychotherapists)	P6
Interventions	11a	Interventions for each group with sufficient detail to allow replication, including how and when they will be administered	P7-10
	11b	Criteria for discontinuing or modifying allocated interventions for a given trial participant (eg, drug dose change in response to harms, participant request, or improving/worsening disease)	P9
	11c	Strategies to improve adherence to intervention protocols, and any procedures for monitoring adherence (eg, drug tablet return, laboratory tests)	NA
	11d	Relevant concomitant care and interventions that are permitted or prohibited during the trial	NA
Outcomes	12	Primary, secondary, and other outcomes, including the specific measurement variable (eg, systolic blood pressure), analysis metric (eg, change from baseline, final value, time to event), method of aggregation (eg, median, proportion), and time point for each outcome. Explanation of the clinical relevance of chosen efficacy and harm outcomes is strongly recommended	P13
Participant timeline	13	Time schedule of enrolment, interventions (including any run-ins and washouts), assessments, and visits for participants. A schematic diagram is highly recommended (see Figure)	P7-14
Sample size	14	Estimated number of participants needed to achieve study objectives and how it was determined, including clinical and statistical assumptions supporting any sample size calculations	P15-16
Recruitment	15	Strategies for achieving adequate participant enrolment to reach target sample size	P7

Methods: Assignment of interventions (for controlled trials)

Allocation:

For peer review only

Sequence generation	16a	Method of generating the allocation sequence (eg, computer- generated random numbers), and list of any factors for stratification. To reduce predictability of a random sequence, details of any planned restriction (eg, blocking) should be provided in a separate document that is unavailable to those who enrol participants or assign	P6-7

For peer review only

Allocation concealment mechanism	16b	Mechanism of implementing the allocation sequence (eg, central telephone; sequentially numbered, opaque, sealed envelopes), describing any steps to conceal the sequence until interventions are assigned	P6-7
Implementation	16c	Who will generate the allocation sequence, who will enrol participants, and who will assign participants to interventions	P6-7
Blinding (masking)	17a	Who will be blinded after assignment to interventions (eg, trial participants, care providers, outcome assessors, data analysts), and how	NA
	17b	If blinded, circumstances under which unblinding is permissible, and procedure for revealing a participant's allocated intervention during the trial	NA
**Methods: Data collection, management, and analysis**			
Data collection methods	18a	Plans for assessment and collection of outcome, baseline, and other trial data, including any related processes to promote data quality (eg, duplicate measurements, training of assessors) and a description of study instruments (eg, questionnaires, laboratory tests) along with their reliability and validity, if known. Reference to where data collection forms can be found, if not in the protocol	P14-15
	18b	Plans to promote participant retention and complete follow-up, including list of any outcome data to be collected for participants who discontinue or deviate from intervention protocols	P14
Data management	19	Plans for data entry, coding, security, and storage, including any related processes to promote data quality (eg, double data entry; range checks for data values). Reference to where details of data management procedures can be found, if not in the protocol	P15
Statistical methods	20a	Statistical methods for analysing primary and secondary outcomes. Reference to where other details of the statistical analysis plan can be found, if not in the protocol	P16-18
	20b	Methods for any additional analyses (eg, subgroup and adjusted analyses)	P17-18
	20c	Definition of analysis population relating to protocol non-adherence (eg, as randomised analysis), and any statistical methods to handle missing data (eg, multiple imputation)	P16

For peer review only

Methods: Monitoring

Data monitoring	21a	Composition of data monitoring committee (DMC); summary of its role and reporting structure; statement of whether it is independent from the sponsor and competing interests; and reference to where further details about its charter can be found, if not in the	P19

For peer review only

	21b	Description of any interim analyses and stopping guidelines, including who will have access to these interim results and make the final decision to terminate the trial	P17-18
Harms	22	Plans for collecting, assessing, reporting, and managing solicited and spontaneously reported adverse events and other unintended effects of trial interventions or trial conduct	P12-13
Auditing	23	Frequency and procedures for auditing trial conduct, if any, and whether the process will be independent from investigators and the sponsor	P19
**Ethics and dissemination**			
Research ethics approval	24	Plans for seeking research ethics committee/institutional review board (REC/IRB) approval	P19,25
Protocol amendments	25	Plans for communicating important protocol modifications (eg, changes to eligibility criteria, outcomes, analyses) to relevant parties (eg, investigators, REC/IRBs, trial participants, trial registries, journals, regulators)	Not provided
Consent or assent	26a	Who will obtain informed consent or assent from potential trial participants or authorised surrogates, and how (see Item 32)	P11-12
	26b	Additional consent provisions for collection and use of participant data and biological specimens in ancillary studies, if applicable	NA
Confidentiality	27	How personal information about potential and enrolled participants will be collected, shared, and maintained in order to protect confidentiality before, during, and after the trial	P19
Declaration of interests	28	Financial and other competing interests for principal investigators for the overall trial and each study site	P24
Access to data	29	Statement of who will have access to the final trial dataset, and disclosure of contractual agreements that limit such access for investigators	P19
Ancillary and post-trial care	30	Provisions, if any, for ancillary and post-trial care, and for compensation to those who suffer harm from trial participation	NA
Dissemination policy	31a	Plans for investigators and sponsor to communicate trial results to participants, healthcare professionals, the public, and other relevant groups (eg, via publication, reporting in results databases, or other data sharing arrangements), including any publication restrictions	P20

For peer review only

31b	Authorship eligibility guidelines and any intended use of professional writers	Not provided
31c	Plans, if any, for granting public access to the full protocol, participant- level dataset, and statistical code	

For peer review only

**Appendices**			
Informed consent materials	32	Model consent form and other related documentation given to participants and authorised surrogates	Not provided
Biological specimens	33	Plans for collection, laboratory evaluation, and storage of biological specimens for genetic or molecular analysis in the current trial and for future [illegible]	NA

*It is strongly recommended that this checklist be read in conjunction with the SPIRIT 2013 Explanation & Elaboration for important clarification on the items. Amendments to the protocol should be tracked and dated. The SPIRIT checklist is copyrighted by the SPIRIT Group under the Creative Commons "Attribution-NonCommercial-NoDerivs 3.0 Unported" license.

For peer review only

BMJ Open

FIRST-line support for Assistance in Breathing in Children (FIRST-ABC): A master protocol of two randomised trials to evaluate the non-inferiority of high flow nasal cannula (HFNC) versus continuous positive airway pressure (CPAP) for non-invasive respiratory support in paediatric critical care

Journal:	*BMJ Open*
Manuscript ID	bmjopen-2020-038002.R1
Article Type:	Protocol
Date Submitted by the Author:	12-Jun-2020
Complete List of Authors:	Richards-Belle, Alvin; Intensive Care National Audit and Research Centre, Clinical Trials Unit<br>Davis, Peter; Bristol Royal Hospital for Children, Paediatric Intensive Care<br>Drikite, Laura; Intensive Care National Audit and Research Centre, Clinical Trials Unit<br>Feltbower, Richard; University of Leeds, Leeds Institute for Data Analytics<br>Grieve, Richard; London School of Hygiene & Tropical Medicine, Department of Health Services Research and Policy<br>Harrison, David; Intensive Care National Audit and Research Centre, Clinical Trials Unit<br>Lester, Julie; Parent representative<br>Morris, Kevin; Birmingham Women's and Children's Hospitals NHS Foundation Trust, Paediatric Intensive Care Unit<br>Mouncey, Paul; Intensive Care National Audit and Research Centre, Clinical Trials Unit<br>Peters, Mark; Great Ormond Street Hospital For Children NHS Trust, Paediatric Intensive Care Unit; University College London, Institute of Child Health<br>Rowan, Kathryn; Intensive Care National Audit and Research Centre, Clinical Trials Unit<br>Sadique, Zia; London School of Hygiene & Tropical Medicine, Department of Health Services Research and Policy<br>Tume, Lyvonne; University of Salford, School of Health and Society<br>Ramnarayan, Padmanabhan; Great Ormond Street Hospital For Children NHS Trust, Children's Acute Transport Service
**Primary Subject Heading**:	Paediatrics
Secondary Subject Heading:	Intensive care, Research methods
Keywords:	Paediatric intensive & critical care < ANAESTHETICS, Paediatric intensive & critical care < INTENSIVE & CRITICAL CARE, Paediatric intensive & critical care < PAEDIATRICS, Clinical trials < THERAPEUTICS,

	STATISTICS & RESEARCH METHODS

I, the Submitting Author has the right to grant and does grant on behalf of all authors of the Work (as defined in the below author licence), an exclusive licence and/or a non-exclusive licence for contributions from authors who are: i) UK Crown employees; ii) where BMJ has agreed a CC-BY licence shall apply, and/or iii) in accordance with the terms applicable for US Federal Government officers or employees acting as part of their official duties; on a worldwide, perpetual, irrevocable, royalty-free basis to BMJ Publishing Group Ltd ("BMJ") its licensees and where the relevant Journal is co-owned by BMJ to the co-owners of the Journal, to publish the Work in this journal and any other BMJ products and to exploit all rights, as set out in our licence.

The Submitting Author accepts and understands that any supply made under these terms is made by BMJ to the Submitting Author unless you are acting as an employee on behalf of your employer or a postgraduate student of an affiliated institution which is paying any applicable article publishing charge ("APC") for Open Access articles. Where the Submitting Author wishes to make the Work available on an Open Access basis (and intends to pay the relevant APC), the terms of reuse of such Open Access shall be governed by a Creative Commons licence – details of these licences and which Creative Commons licence will apply to this Work are set out in our licence referred to above.

Other than as permitted in any relevant BMJ Author's Self Archiving Policies, I confirm this Work has not been accepted for publication elsewhere, is not being considered for publication elsewhere and does not duplicate material already published. I confirm all authors consent to publication of this Work and authorise the granting of this licence.

For peer review only

+FIRST-line support for Assistance in Breathing in Children (FIRST-ABC): A master protocol of two randomised trials to evaluate the non-inferiority of high flow nasal cannula (HFNC) versus continuous positive airway pressure (CPAP) for non-invasive respiratory support in paediatric critical care

AUTHORS

Alvin Richards-Belle[1], Peter Davis[2], Laura Drikite[1], Richard Feltbower[3], Richard D. Grieve[4], David A. Harrison[1], Julie Lester[5], Kevin P. Morris[6], Paul R. Mouncey[1], Mark J. Peters[7,8,10], Kathryn M. Rowan[1], M. Zia Sadique[4], Lyvonne N. Tume[9], Padmanabhan Ramnarayan[10,11]

Affiliations

[1]Clinical Trials Unit, Intensive Care National Audit & Research Centre (ICNARC), Napier House, 24 High Holborn, London, WC1V 6AZ, United Kingdom

[2]Paediatric Intensive Care, Bristol Royal Hospital for Children, University Hospitals Bristol NHS Foundation Trust, Upper Maudlin Street, Bristol, BS2 8BJ, United Kingdom

[3]Leeds Institute for Data Analytics, University of Leeds, School of Medicine, Worsley Building, Leeds, LS2 9JT, United Kingdom

[4]Department of Health Services Research and Policy, London School of Hygiene & Tropical Medicine, 15-17 Tavistock Place, London, WC1H 9SH, United Kingdom

[5]Parent representative, United Kingdom

[6]Paediatric Intensive Care, Birmingham Children's Hospital, Birmingham Women's and Children's NHS Foundation Trust, Steelhouse Lane, Birmingham, B4 6NH, United Kingdom

[7]Paediatric Intensive Care Unit, Great Ormond Street Hospital for Children NHS Foundation Trust, and NIHR Biomedical Research Centre, WC1N 3JH, London, United Kingdom

[8]Respiratory Critical Care and Anaesthesia Unit, UCL Great Ormond Street Institute of Child Health, London, WC1N 1EH, United Kingdom

[9]School of Health & Society, University of Salford, Mary Seacole Building, Frederick Road Campus, Broad St, Frederick Road Campus, Salford, M6 6PU, United Kingdom

[10]Children's Acute Transport Service, Great Ormond Street Hospital for Children NHS Foundation Trust and NIHR Biomedical Research Centre, WC1N 3JH, London, United Kingdom

[11]Paediatric Intensive Care Unit, St Mary's Hospital, Imperial College Healthcare NHS Trust, Praed Street, Paddington, London, W2 1NY, United Kingdom

For peer review only

Corresponding author

Dr Padmanabhan Ramnarayan

Consultant in Paediatric Intensive Care & Retrieval

Children's Acute Transport Service (CATS)

Heart and Lung Directorate

Great Ormond Street Hospital for Children NHS Foundation Trust

26-27 Boswell Street

London WC1N 3JZ

United Kingdom

Telephone: 020 7430 5850

Email: p.ramnarayan@gosh.nhs.uk

Keywords

non-invasive respiratory support, high flow nasal cannula, continuous positive airway pressure, randomised clinical trial

Word count: Abstract (298), Main manuscript (4483)

For peer review only

For peer review only - http://bmjopen.bmj.com/site/about/guidelines.xhtml

Abstract

Introduction

Even though respiratory support is a common intervention in paediatric critical care, there is no randomised controlled trial (RCT) evidence regarding the effectiveness of two commonly used modes of non-invasive respiratory support (NRS), continuous positive airway pressure (CPAP) and high-flow nasal cannula therapy (HFNC). FIRST-ABC is a master protocol of two pragmatic non-inferiority RCTs to evaluate the clinical and cost-effectiveness of HFNC (compared to CPAP) as the first-line mode of support in critically ill children.

Methods and analysis

We will recruit participants over a 30-month period at 25 UK paediatric critical care units (paediatric intensive care units/high dependency units). Patients are eligible if admitted/accepted for admission, aged >36 weeks corrected gestational age and <16 years, and assessed by the treating clinician to require non-invasive respiratory support for an acute illness (step-up RCT) or within 72 hours of extubation following a period of invasive ventilation (step-down RCT). Due to the emergency nature of the treatment, written informed consent will be deferred to after randomisation. Randomisation will occur 1:1 to CPAP or HFNC, stratified by site and age (<12 vs. ≥12 months). The primary outcome is time to liberation from respiratory support for a continuous period of 48 hours. A total sample size of 600 patients in each RCT will provide 90% power with a type I error rate of 2.5% (one-sided) to exclude the pre-specified noninferiority margin of hazard ratio of 0.75. Primary analyses will be undertaken separately in each RCT in both the intention-to-treat and per-protocol populations.

Ethics and dissemination

This master protocol received favourable ethical opinion from NHS East of England - Cambridge South Research Ethics Committee (reference: 19/EE/0185) and approval from the Health Research Authority (reference: 260536). Results will be disseminated via publications in peer reviewed medical journals and presentations at national and international conferences.

Trial registration

ISRCTN60048867; Pre-results.

For peer review only

For peer review only - http://bmjopen.bmj.com/site/about/guidelines.xhtml

Article Summary

Strengths and limitations of this study

1. FIRST-ABC is a master protocol of the two largest RCTs to date to study the clinical and cost-effectiveness of high flow nasal cannula as the first-line mode of non-invasive respiratory support in critically ill children.

2. The FIRST-ABC master protocol includes two separate RCTs, one in acutely ill children requiring respiratory support (step-up RCT) and one in children requiring respiratory support after extubation from invasive ventilation (step-down RCT), to address the research question in two distinct but common clinical scenarios.

3. The design and conduct of FIRST-ABC has been informed by a successful pilot RCT that confirmed the feasibility of delivering a large pragmatic trial in critically ill children.

4. The choice of the primary outcome, time to liberation from all forms of respiratory support for a continuous period of at least 48 hours, was informed by clinicians as well as through patient and public involvement.

5. Changes to clinical practice during the trial period, and a resultant shift in equipoise regarding the choice of first-line mode of respiratory support in critically ill children, may affect the ability to recruit successfully to the RCTs.

For peer review only

For peer review only - http://bmjopen.bmj.com/site/about/guidelines.xhtml

Introduction

Nearly 75% of the 20,000 critically ill children admitted annually to United Kingdom (UK) paediatric intensive care units (PICUs) receive some form of respiratory support.[1] Increasing recognition of the risks of invasive ventilation has prompted greater use of non-invasive respiratory support (NRS) worldwide.[1 2] Two main modes of NRS are used, to support acutely ill children with respiratory failure or to provide post-extubation support after a spell of invasive ventilation.

Continuous positive airway pressure (CPAP) has been used by PICUs for over three decades.[3-5] Although observational data suggest that CPAP is effective, there have been few randomised clinical trials (RCTs) of CPAP in critically ill children.[5-7] CPAP can be uncomfortable and may be associated with complications such as air-leak and nasal trauma, often necessitating the use of sedation, close monitoring and a high level of nursing input. An alternate mode of NRS, high flow nasal cannula (HFNC), has gained popularity more recently. It appears easy to use and is well-tolerated.[8-11] Between 16 and 35% of PICU admissions receive HFNC at some point during their stay.[1 12 13] The potential benefits of HFNC (improved patient comfort, safety profile and ease of nursing care), must be balanced against its potential risks (air leak, abdominal distension and nosocomial infection), and concerns regarding unnecessary prolongation of PICU/hospital stay and excess mortality from delayed escalation. There are few RCTs comparing HFNC with CPAP in the PICU setting. Previous RCTs do not include children with a range of ages and diagnoses needing either *step-up* or *step-down* (post-extubation) care, making it impossible to generalise their findings to contemporary practice.[14-16]

FIRST-ABC therefore addresses an important clinical dilemma faced daily by critical care clinicians: in a child requiring NRS, which modality, HFNC or CPAP, should they use as first-line therapy to achieve the best patient outcomes? Our research question was prioritised by clinicians as well as parents/patients. We previously successfully completed a pilot RCT, which supported the feasibility of performing a large pragmatic RCT comparing CPAP and HFNC in critically ill children, and informed its design and conduct.[17] This protocol has been written in accordance with the Standard Protocol Items: Recommendations for Interventional Trials (SPIRIT) statement.[18]

For peer review only

For peer review only - http://bmjopen.bmj.com/site/about/guidelines.xhtml

Methods

Hypothesis

In critically ill children assessed by the treating clinician to require non-invasive respiratory support (NRS), first-line use of high flow nasal cannula (HFNC) is non-inferior to continuous positive airway pressure (CPAP) in time to liberation from respiratory support.

Aim

To evaluate the clinical and cost-effectiveness of the use of HFNC, as compared with CPAP, when used as the first-line mode in critically ill children requiring NRS:

A. for an acute illness (*step-up RCT*);
B. within 72 hours of extubation following a period of invasive ventilation (*step-down RCT*).

Primary objective

To evaluate the non-inferiority of HFNC, as compared with CPAP, when used as the first-line mode of NRS, both as a step-up treatment (*step-up RCT*) and as a step-down treatment (*step-down RCT*), on the time to liberation from respiratory support.

Design

FIRST-ABC is a master protocol comprising two pragmatic, multi-centre, parallel groups, non-inferiority RCTs (*step-up RCT* and *step-down RCT*) with shared infrastructure, including an internal pilot stage and integrated health economic evaluation. This design allows the research question to be addressed in each of the two important populations in an efficient way by minimising time and infrastructure costs as compared with conducting two sequential RCTs.[19] The pragmatic study design ensures that research findings can be more easily generalised to real-world practice.

A non-inferiority design was chosen based on previous RCTs in this area and feedback from clinicians from the UK Paediatric Intensive Care Society – Study Group in July 2017 which indicated that the potential benefits of HFNC (in terms of patient comfort and ease of use) would mean that it would likely be preferred in usual practice even if not shown to be superior to CPAP.

Setting

FIRST-ABC is set in NHS paediatric critical care units (PICU and/or high dependency units (HDUs)) across England, Wales and Scotland. General medical-surgical, cardiac and mixed units were considered for participation. Sites are eligible to take part if they confirm collective

For peer review only - http://bmjopen.bmj.com/site/about/guidelines.xhtml

equipoise regarding the choice of first-line NRS in their unit and commit to following trial procedures, including randomisation and data collection. Sites can start recruitment only after a site initiation visit and all relevant regulatory approvals.

Population

Critically ill children assessed by the treating clinician to require NRS for (A) an acute illness (*step-up RCT*) or (B) within 72 hours of extubation following a period of invasive ventilation (*step-down RCT*).

Screening

Potentially eligible patients admitted/accepted for admission to the participating critical care unit will be screened against the inclusion/exclusion criteria by the local clinical/research team. For the step-up RCT, all admissions to the unit will be screened. For the step-down RCT, all patients extubated during unit admission will be screened. From these, Screening and Enrolment Logs will record enrolled patients, reasons for exclusion and reasons eligible patients are not enrolled.

<u>Inclusion criteria</u>

1) Admitted/Accepted for admission to PICU/HDU
2) Age >36 weeks corrected gestational age and <16 years
3) Assessed by the treating clinician to require non-invasive respiratory support, <u>EITHER</u>
    A. for an acute illness (*step-up RCT*) <u>OR</u>
    B. within 72 hours of extubation following a period of invasive ventilation (*step-down RCT*).

<u>Exclusion criteria</u>

1) Assessed by the treating clinician to require immediate intubation and invasive ventilation due to severe hypoxia, acidosis and/or respiratory distress, upper airway obstruction, inability to manage airway secretions or recurrent apnoeas
2) Tracheostomy in place
3) Received HFNC/CPAP for >2 hours in the prior 24 hours
4) On home non-invasive ventilation prior to PICU/HDU admission
5) Presence of untreated air-leak (pneumothorax/pneumomediastinum)
6) Midfacial/craniofacial anomalies (unrepaired cleft palate, choanal atresia) or recent craniofacial surgery
7) Agreed ‘not for intubation’ or other limitation of critical care treatment plan in place.

For peer review only - http://bmjopen.bmj.com/site/about/guidelines.xhtml

8) Previously recruited to FIRST-ABC (step-up RCT or step-down RCT on this or a previous admission)
9) Clinician decision to start other form of non-invasive respiratory support (i.e. not HFNC or CPAP - e.g. bilevel positive pressure and negative pressure ventilation)

Randomisation

Randomisation will be performed after confirming eligibility and as close as possible to the anticipated start of the randomised treatment. In each RCT, eligible patients will be randomised in a 1:1 ratio to either CPAP or HFNC using a central telephone/web-based randomisation service available 24 hours/7 days a week. The randomisation sequence will be computer generated and variable block sizes will be used to strengthen allocation concealment. Randomisation will be stratified by site and age (<12 months versus ≥12 months) to minimise imbalance arising from unit practices and interface selection.

The randomised treatment will be commenced as soon as practically possible. Following randomisation, each participant will be assigned a unique FIRST-ABC Trial Number and a Case Report Form (CRF) completed by the local research team.

Delivery of HFNC

Any approved medical device capable of delivering heated, humidified, high flow through nasal cannulae can be used to provide HFNC at the prescribed gas flow rates during the trial period. To standardise treatment, clinical criteria and guidance for the initiation, maintenance and weaning of HFNC are provided in a trial algorithm (**Error! Reference source not found.**). The trial algorithms were developed iteratively in consultation with paediatric critical care clinicians across the UK (both via email and in person at a Collaborators' Meeting held prior to the start of the trial).

The trial recommends that patients are assessed for response to the treatment, readiness to wean and for stopping HFNC, as per the HFNC algorithm, at least twice per day (e.g. at ward rounds).

Delivery of CPAP

CPAP will be started using an approved medical device at a set expiratory pressure of 7-8 cm H_2O. The trial does not specify any particular device or patient interface for the provision of

For peer review only - http://bmjopen.bmj.com/site/about/guidelines.xhtml

CPAP. To standardise treatment, clinical criteria and guidance for the initiation, maintenance and weaning of CPAP are provided in a trial algorithm (**Error! Reference source not found.**). It is recommended that patients are assessed for response to the treatment, readiness to wean and for stopping CPAP, as per the CPAP algorithm, at least twice per day (e.g. at ward rounds).

Clinical practice during the trial

Since staff in participating sites already use HFNC and CPAP, no additional central training related to the use of HFNC or CPAP will be provided for the trial, but resources for training in the trial algorithms will be provided. As the medical devices and interfaces that deliver HFNC and CPAP are easily distinguishable from each other, it will not be possible to blind the patient, parents/guardians or clinical staff.

The trial algorithms will be followed until the patient has been liberated from all forms of respiratory support for at least 48 continuous hours. As per current practice, clinicians will be able to stop HFNC/CPAP and switch to the other treatment or escalate to other forms of respiratory support, if clinically deemed necessary. Pre-specified objective criteria to identify non-responders to HFNC/CPAP are provided in the algorithms as a guide for clinicians considering switching or escalating respiratory support. Reasons for switches or escalations will be recorded. Patients who switch or escalate treatments will remain in the trial and continue to be monitored until liberation from respiratory support. All other usual care (e.g. sedation, feeding) will be at the discretion of the treating clinical team.

Consent procedures

Consent will be sought for the child (patient) from their parent/legal guardian. Children become eligible for FIRST-ABC when critically ill, a profoundly stressful time for parents/guardians, during which there are ethical concerns both about the burden of trying to understand the trial and the ability to provide informed consent. Initiation of NRS typically occurs during a time-sensitive situation, where delays could be detrimental to the child and to the trial's scientific validity. Moreover, both CPAP and HFNC are already widely used in standard practice across the NHS. Considering these reasons, FIRST-ABC has been given ethical approval to use a deferred consent model ('research without prior consent'). Once a patient is confirmed eligible, they will be randomised and the allocated treatment (CPAP or HFNC) commenced as soon as possible. This model, developed in line with the CONseNt methods in paediatric Emergency and

For peer review only - http://bmjopen.bmj.com/site/about/guidelines.xhtml

urgent Care Trials (CONNECT) guidance,[20] has been found acceptable to parents/guardians and clinicians in several recent RCTs in the PICU setting[17 21-25] and is informed by experience/feedback from the pilot RCT.[17]

Following randomisation, a trained, delegated member of the local research team will approach the child's parents/guardians as soon as appropriate and practically possible to discuss the trial (usually within 24-48 hours of randomisation). A Participant Information Sheet (PIS) will be provided, covering information about the purpose of the trial; the consequences of participating or not; confidentiality; use of personal data; data security; and the future availability of the trial results. A Consent Form (see supplementary file 1) will be provided, indicating that: the information given has been read and understood; participation is voluntary and consent can be withdrawn at any time without consequence; and that consent is given for access to medical records to continue data collection, to receive a follow-up questionnaire and for anonymised data to be shared in the future. Due to age and severity of illness, it will not be possible to involve the patient in the consenting process. Instead, assent will be obtained prior to hospital discharge if their condition allows (e.g. they regain mental capacity).

A modification of the consent procedure will be utilised for two rare situations where either the patient: a) is discharged from hospital prior to obtaining consent, or b) dies prior to consent being sought.[24 26] In the former, the local research team will follow up with the parent/guardian, initially by phone and then by post, for consent. Postal contact will be made again if there is no response after four weeks. If no Consent Form is received within four weeks of the second letter, the participant will be included in the trial unless they notify the research team otherwise. In the latter situation, the local research team will obtain information from colleagues and bereavement counsellors to establish the most appropriate clinical/research team member to notify the parents/guardians of involvement in the trial. If approach for consent is deemed not appropriate prior to the parent/guardian's departure from hospital, then they will be approached by post four weeks post-randomisation. The letter will explain how to opt out of the trial. Postal contact will be made again if there is no response after four weeks. If no Consent Form is received within four weeks of the second letter, the participant's data will be included in the trial.

If informed consent is refused or withdrawn, this decision will be respected and abided by, and no further contact made. All data occurring up to the point of this decision will be retained in the trial, unless parents/guardians request otherwise.

For peer review only - http://bmjopen.bmj.com/site/about/guidelines.xhtml
For peer review only

Safety monitoring

Adverse Event (AE) reporting will follow the Health Research Authority (HRA) guidelines on safety reporting in studies which do not use Investigational Medicinal Products (non-CTIMPs). The following events have been pre-specified as potential AEs that could be related to CPAP and/or HFNC and observed in participants from the date and time of randomisation until 48 hours of liberation from all forms of respiratory support:

1. Nasal trauma
2. Facial/neck trauma
3. Abdominal distension
4. Pneumothorax
5. Pneumomediastinum
6. Subcutaneous emphysema
7. Facial thermal injury
8. Respiratory arrest
9. Cardiac arrest
10. Aspiration

Occurrences of the specified, expected adverse events will be recorded for all randomised patients. Considering that eligible patients are critically ill and at increased risk of experiencing AEs, occurrences of non-specified, adverse events will only be reported if considered to be related to either CPAP or HFNC (i.e. 'possibly', 'probably' or 'definitely' related). Any event classified as 'severe' or 'life-threatening' in severity is considered a Serious Adverse Event (SAE) and must be reported to ICNARC CTU. If the SAE is evaluated by the Trial Management Group (TMG) as a related and unexpected SAE, the ICNARC CTU will submit a report to the REC within 15 calendar days.

Questionnaire follow-up

At six months, after assessing the child's survival status, each consenting parent will be sent a questionnaire (via email or post) by the ICNARC CTU to assess Health-related Quality of Life (HrQoL) and health service/resource use. Non-responders will be followed-up by telephone three weeks later.

For peer review only - http://bmjopen.bmj.com/site/about/guidelines.xhtml
For peer review only

Outcome measures

Primary outcome

Time to liberation from respiratory support, defined as the start of a 48-hour period during which the child was free of all forms of respiratory support.

The primary outcome definition of respiratory support does not include administration of supplementary oxygen alone. In addition, the primary outcome will to be monitored/recorded after discharge from critical care, as necessary. We chose time to liberation from respiratory support, instead of rate of (re)intubation, as the primary outcome for several reasons, including: 1) through our patient and public involvement (PPI) work, parents/families reported that even though intubation was clearly an undesirable outcome, the fact that the child needed a 'breathing machine' of any description would be more important for them, in terms of assessing the success or failure of the intervention. Normalisation of 'breathing' was an important outcome prioritised over intubation; 2) since the rate of intubation on average was around 20% in the pilot RCT, nearly 80% of patients may not fulfil the intubation outcome. In these patients, several non-invasive support modes may be used, which prolong the time the patient is on 'breathing support' with resource implications for critical care. Clinicians felt that it was important that the effect of the intervention was assessed on patients who did not need intubation as well as on those who did. 3) unpublished data from the pilot RCT showed that the length of respiratory support is longer in patients who need intubation compared to those who do not. Therefore, the adverse impact of intubation is likely reflected in longer duration of respiratory support.

Secondary outcomes

- Mortality at PICU/HDU discharge, day 60 and day 180
- Rate of (re)intubation at 48 hours
- Duration of PICU/HDU and hospital stay
- Patient comfort, during randomised treatment and during non-invasive respiratory support (i.e. HFNC and/or CPAP), assessed using the COMFORT-B score[27]
- Proportion of patients in whom sedation is used during non-invasive respiratory support
- Parental stress, in hospital at/around the time of consent at 24-48 hours, measured using the Parental Stressor Scale: PICU[28]
- HrQoL at six months using age-appropriate Pediatric Quality of Life Inventory (Peds-QL)[29] and Child Health Utility (CHU-9D) questionnaire[30]

For peer review only - http://bmjopen.bmj.com/site/about/guidelines.xhtml

Cost effectiveness analysis (CEA) outcomes

- Total costs at six months
- Quality-Adjusted Life-Years (QALYs) at six months
- Incremental net monetary benefit gained at a willingness-to-pay of £20,000 per QALY at six months associated with HFNC versus CPAP[31]

Data collection

To maximise efficiency, FIRST-ABC collaborates with the Paediatric Intensive Care Audit Network (PICANet) to make best use of established PICU data collection infrastructure. Where possible, recruited patients will be consented for data linkage with routine sources (e.g. national death registration data via NHS Digital or equivalent). Additional trial-specific data collection items are limited to the minimum required to deliver trial objectives (**Error! Reference source not found.**).

For peer review only

For peer review only - http://bmjopen.bmj.com/site/about/guidelines.xhtml

Table 1 Patient data collection schedule

	Baseline	At time of consent	During non-invasive respiratory support	End of PICU/HDU stay	End of hospital stay	At six months
**In-hospital**						
Clinical/baseline data	✓					
Patient/parent details		✓				
Types of respiratory support received*	✓		✓			
Patient comfort and sedation use			✓			
Parental stress		✓				
Discharge data				✓	✓	
Safety monitoring data			✓			
**At follow-up**						
PedsQL						✓
CHU-9D						✓
Health services/ resource use						✓

PICU: paediatric intensive care unit, HDU: high dependency unit, PedsQL: Pediatric Quality of Life Inventory (Peds-QL), CHU-9D: Child Health Utility questionnaire.

* *including weaning, switches and escalations from High Flow Nasal Cannula (HFNC)/Continuous Positive Airway Pressure (CPAP)*

For peer review only

All participant data will be entered onto the secure electronic case report form and undergo validation checks for completeness, accuracy and consistency. The site Principal Investigator will oversee and be responsible for data collection, quality and recording.

Statistical Methods

Sample size

To achieve 90% power with a type I error rate of 2.5% (one-sided) to exclude the pre-specified noninferiority margin of HR=0.75 requires 508 events to be observed. Based on pilot RCT

For peer review only - http://bmjopen.bmj.com/site/about/guidelines.xhtml

data,[17] we anticipate 5% censoring due to death or transfer, leading to a required sample size of 268 patients per group in each of the two RCTs. To allow for withdrawal/refusal of consent, and for exclusion due to non-adherence in the per-protocol (PP) population, we will recruit a total sample size of 600 patients in each RCT.

Internal pilot

Data will be analysed at the end of the internal pilot stage (months 7-12 of the grant timeline) on patients recruited during the first six months in each RCT. The RCTs will progress from pilot to full trial based on pre-specified progression criteria related to successful site set-up, screening and recruitment, and adherence. The final decision on progression will be made by the funder after recommendation, or not, by the Trial Steering Committee (TSC).

Clinical effectiveness analysis

All analyses will be publicly lodged[32] in a statistical analysis plan, a priori, before the investigators are unblinded to any trial outcomes. Following best practice for non-inferiority trials, the primary analyses will be undertaken in both intention-to-treat (ITT) and PP populations, with robust conclusions possible in the situation where both populations provide concordant results. Results will be reported in accordance with the CONSORT statement extensions for non-inferiority and pragmatic trials.[33 34]

Analyses will be undertaken independently for each RCT. In each RCT, baseline patient characteristics will be compared between the two groups to observe balance and the success of randomisation. These comparisons will not be subjected to statistical testing. The delivery of the intervention will be described for each group in detail, including (but not limited to) number and percentage of patients who commence the randomised treatment, remain on the randomised treatment until liberation from ventilation, who are changed to a different mode of respiratory support.

HFNC will be considered non-inferior to CPAP if the lower bounds of the 95% confidence intervals for the hazard ratio (HR) from Cox regression models on time to liberation from respiratory support fitted in both the ITT and PP populations exclude the pre-specified non-inferiority margin of 0.75 (corresponding to approximately a 16-hour increase in median time to liberation, based on pilot RCT data). This margin was considered adequate such that the other potential benefits of HFNC in terms of comfort and tolerability would mean that it would be likely to be preferred in usual practice. The Cox regression models will be adjusted for important

For peer review only - http://bmjopen.bmj.com/site/about/guidelines.xhtml

baseline characteristics. The covariates for inclusion in the regression models will be selected a priori based on an established relationship with outcome for critically ill children, and not because of observed imbalance, significance in univariable analyses or by a stepwise selection method.

Subgroup analyses for the primary outcome will be performed to test for interactions between the effect of allocated treatment group and the following baseline covariates:

- age (<12 months versus ≥12 months)
- severity of respiratory distress at randomisation (severe versus mild/moderate)
- Co-morbidities (None versus Neurological/neuromuscular versus Other)
- Sp02/Fi02 (SF) ratio at randomisation
- for step-up RCT only:
    - clinical indication (bronchiolitis versus other respiratory (airway problem, asthma/wheeze or any other respiratory) versus cardiac versus other (neurological, sepsis/infection, any other))
    - whether child was on NRS at randomisation (Yes/No)
- for step-down RCT only:
    - length of prior invasive mechanical ventilation (<5 days versus ≥5 days)
    - reason for invasive mechanical ventilation (cardiac versus other)
    - planned (randomisation followed by extubation) vs rescue (extubation followed by randomisation) non-invasive respiratory support

We will treat age as a continuous variable and determine whether the model goodness-of-fit is better versus treating age as a categorical term for any analyses focusing on those over the age of 12 months. We anticipate a high proportion of patients will be aged <12 months and therefore exploration of age effects in the older ages will only be conducted if there are sufficient patient numbers.

As a sensitivity analysis, the primary analysis will be repeated using time to start weaning of NRS (i.e. duration of 'acute' respiratory support) and time to meeting objective 'readiness to wean NRS' criteria.

Secondary analyses of binary outcomes (mortality, reintubation) will be performed by Fisher's exact test and adjusted logistic regression. Duration of survival to day 180 will be plotted as

For peer review only - http://bmjopen.bmj.com/site/about/guidelines.xhtml

Kaplan-Meier survival curves, compared unadjusted with the log rank test and adjusted using Cox regression models. Analyses of duration of PICU/HDU and hospital stay will be performed by Wilcoxon rank-sum tests, stratified by survival status. Analyses of COMFORT-B score, sedation use, PSS:PICU and HrQoL will be performed by t-tests and adjusted linear regression.

In the step-up RCT, a single interim analysis will be carried out after the recruitment and follow-up to day 60 of 300 patients. The interim analysis will use a Peto-Haybittle stopping rule to recommend early termination due to superiority of either intervention ($P<0.001$) in time to liberation from respiratory support or evidence of harm from either intervention ($P<0.05$) in mortality at day 60. Both tests will be performed using a log-rank test on all available data within the ITT population. Further interim analyses will be performed only if requested by the Data Monitoring and Ethics Committee (DMEC).

In the step-down RCT, due to faster than anticipated recruitment, no formal interim analysis will be performed. Safety data (counts and percentages of adverse events by arm, and a line listing of SAEs) will be available for scrutiny by the DMEC, by the end of the internal pilot stage.

Integrated health economic evaluation

The CEA will take an NHS and Personal Social Services perspective.[31] Patient-level resource use data will be obtained from CRFs, PICANet, and a parent-completed health services questionnaire (HSQ). Resource use data from the PICU/HDU stay will be taken from the CRF and linked routine data from PICANet. Information on subsequent PICU/HDU and hospital admissions will be obtained via data linkage with PICANet and NHS Digital and also through completion of the HSQ. Data on the level of care for PICU bed-days will be gathered through routine collection of the Paediatric Critical Care Minimum Dataset (PCCMDS) in the participating sites via the PICANet database. Use of primary care and community health services will be assessed by HSQ at six months. Patient-level resource use data will be combined with appropriate unit costs from the NHS payment by results and Personal Social Services Research Unit databases to report total costs per patient for up to six months post-randomisation. Data from PedsQL and CHU-9D at six months will be combined with survival data to report QALYs at six months. The CEA will follow the intention-to-treat principle and report the mean (95% confidence interval) incremental costs, QALYs and net monetary benefit at six months. The CEA will use multilevel linear regression models that allow for clustering of patients at site. The analysis will adjust for key baseline covariates at both patient and site level.

For peer review only - http://bmjopen.bmj.com/site/about/guidelines.xhtml

Ethics and dissemination

Research ethics

The trial received favourable ethical opinion from NHS East of England - Cambridge South Research Ethics Committee (reference number: 19/EE/0185) and approval from the HRA (Integrated Research Application System (IRAS) number: 260536). Evidence of local confirmation of capacity and capability at each site must be provided to the ICNARC CTU prior to site activation.

Confidentiality

ICNARC CTU will act to preserve participant confidentiality and will not disclose or reproduce any information by which participants could be identified. All data will be stored securely.

Oversight

The TMG, led by the Chief Investigator, is responsible for the management of FIRST-ABC. It meets regularly and includes the Investigators and ICNARC CTU trial team. FIRST-ABC is managed by the ICNARC CTU in accordance with the Medical Research Council's Good Research Practice: Principles and Guidelines[35] which is based on the International Conference on Harmonisation guidelines on Good Clinical Practice[36] principles and the UK Department of Health's Policy Framework for Health and Social Care Research.[37] The on-site monitoring plan will follow a risk-based strategy.

A majority independent TSC has been established to monitor trial progress and includes PPI representatives, experienced clinicians and researchers/statisticians, in addition to the Chief Investigator and Head of Research at ICNARC. An independent DMEC, comprising experienced clinicians and statisticians, has been established to monitor patient recruitment and retention, adherence and safety.

The Great Ormond Street Hospital for Children NHS Foundation Trust are the trial sponsor (reference: 17IA05). As the sponsor is an NHS organisation, NHS indemnity will apply for legal liability arising from the design, management and conduct of the research.

Patient and Public Involvement (PPI)

We had considerable PPI input into the pilot RCT[17] as well as the main trial described here. Following the pilot RCT, the PPI Group for Research at Great Ormond Street Hospital was consulted on the choice of the primary outcome for the main RCTs (see *Outcome measures*

For peer review only - http://bmjopen.bmj.com/site/about/guidelines.xhtml

section). The parent of a child who received respiratory support is a co-investigator and has actively contributed to the trial design and procedures, including the use of deferred consent and patient/parent information sheets and other materials.

Trial status

This paper presents the master protocol (v1.2, dated 23 January 2020)[38] for the two largest RCTs studying the clinical and cost effectiveness of HFNC therapy as the first-line mode of NRS in critically ill children. It will provide robust evidence for the two distinct but common clinical scenarios in which NRS is primarily used. The first participant was recruited in August 2019. At the time of submission, patient recruitment was ongoing – with recruitment planned to complete in November 2020 and January 2022 for the step-down RCT and step-up RCT, respectively. Each RCT will be disseminated independently, including through publication in peer-reviewed medical journals and at national and international conferences.

For peer review only

For peer review only - http://bmjopen.bmj.com/site/about/guidelines.xhtml

References

1. Paediatric Intensive Care Audit Network (PICANet). Annual Report 2019. Leeds: Universities of Leeds and Leicester, 2019.
2. Wolfler A, Calderini E, Iannella E, et al. Evolution of Noninvasive Mechanical Ventilation Use: A Cohort Study Among Italian PICUs. *Pediatr Crit Care Med* 2015;16(5):418-27. doi: 10.1097/PCC.0000000000000387 [published Online First: 2015/04/02]
3. Shah PS, Ohlsson A, Shah JP. Continuous negative extrathoracic pressure or continuous positive airway pressure compared to conventional ventilation for acute hypoxaemic respiratory failure in children. *Cochrane Database Syst Rev* 2013(11):CD003699. doi: 10.1002/14651858.CD003699.pub4 [published Online First: 2013/11/05]
4. Jat KR, Mathew JL. Continuous positive airway pressure (CPAP) for acute bronchiolitis in children. *Cochrane Database Syst Rev* 2015;1:CD010473. doi: 10.1002/14651858.CD010473.pub2 [published Online First: 2015/01/08]
5. Donlan M, Fontela PS, Puligandla PS. Use of continuous positive airway pressure (CPAP) in acute viral bronchiolitis: a systematic review. *Pediatr Pulmonol* 2011;46(8):736-46. doi: 10.1002/ppul.21483 [published Online First: 2011/05/28]
6. Thia LP, McKenzie SA, Blyth TP, et al. Randomised controlled trial of nasal continuous positive airways pressure (CPAP) in bronchiolitis. *Arch Dis Child* 2008;93(1):45-7. doi: 10.1136/adc.2005.091231 [published Online First: 2007/03/09]
7. Peters MJ, Agbeko R, Davis P, et al. Randomized Study of Early Continuous Positive Airways Pressure in Acute Respiratory Failure in Children With Impaired Immunity (SCARF) ISRCTN82853500. *Pediatric critical care medicine : a journal of the Society of Critical Care Medicine and the World Federation of Pediatric Intensive and Critical Care Societies* 2018;19(10):939-48. doi: 10.1097/PCC.0000000000001683
8. Lee JH, Rehder KJ, Williford L, et al. Use of high flow nasal cannula in critically ill infants, children, and adults: a critical review of the literature. *Intensive Care Med* 2013;39(2):247-57. doi: 10.1007/s00134-012-2743-5
9. Spentzas T, Minarik M, Patters AB, et al. Children with respiratory distress treated with high-flow nasal cannula. *J Intensive Care Med* 2009;24(5):323-8. doi: 10.1177/0885066609340622 [published Online First: 2009/08/26]
10. Ramnarayan P, Schibler A. Glass half empty or half full? The story of high-flow nasal cannula therapy in critically ill children. *Intensive Care Med* 2017;43(2):246-49. doi: 10.1007/s00134-016-4663-2 [published Online First: 2017/01/27]
11. Morris JV, Kapetanstrataki M, Parslow RC, et al. Patterns of Use of Heated Humidified High-Flow Nasal Cannula Therapy in PICUs in the United Kingdom and Republic of Ireland*. *Pediatric Critical Care Medicine* 2019;20(3):223-32. doi: 10.1097/pcc.0000000000001805
12. Coletti KD, Bagdure DN, Walker LK, et al. High-Flow Nasal Cannula Utilization in Pediatric Critical Care. *Respir Care* 2017;62(8):1023-29. doi: 10.4187/respcare.05153 [published Online First: 2017/06/08]
13. Baudin F, Gagnon S, Crulli B, et al. Modalities and Complications Associated With the Use of High-Flow Nasal Cannula: Experience in a Pediatric ICU. *Respir Care* 2016;61(10):1305-10. doi: 10.4187/respcare.04452 [published Online First: 2016/08/04]

For peer review only

For peer review only - http://bmjopen.bmj.com/site/about/guidelines.xhtml

14. Mayfield S, Jauncey-Cooke J, Hough JL, et al. High-flow nasal cannula therapy for respiratory support in children. *Cochrane Database Syst Rev* 2014(3):Cd009850. doi: 10.1002/14651858.CD009850.pub2 [published Online First: 2014/03/08]
15. Beggs S, Wong ZH, Kaul S, et al. High-flow nasal cannula therapy for infants with bronchiolitis. *Cochrane Database Syst Rev* 2014;1:CD009609. doi: 10.1002/14651858.CD009609.pub2 [published Online First: 2014/01/21]
16. Milesi C, Essouri S, Pouyau R, et al. High flow nasal cannula (HFNC) versus nasal continuous positive airway pressure (nCPAP) for the initial respiratory management of acute viral bronchiolitis in young infants: a multicenter randomized controlled trial (TRAMONTANE study). *Intensive Care Med* 2017;43(2):209-16. doi: 10.1007/s00134-016-4617-8 [published Online First: 2017/01/27]
17. Ramnarayan P, Lister P, Dominguez T, et al. FIRST-line support for Assistance in Breathing in Children (FIRST-ABC): a multicentre pilot randomised controlled trial of high-flow nasal cannula therapy versus continuous positive airway pressure in paediatric critical care. *Crit Care* 2018;22(1):144. doi: 10.1186/s13054-018-2080-3 [published Online First: 2018/06/06]
18. Chan AW, Tetzlaff JM, Altman DG, et al. SPIRIT 2013 statement: defining standard protocol items for clinical trials. *Ann Intern Med* 2013;158(3):200-7. doi: 10.7326/0003-4819-158-3-201302050-00583 [published Online First: 2013/01/09]
19. Woodcock J, LaVange LM. Master Protocols to Study Multiple Therapies, Multiple Diseases, or Both. *N Engl J Med* 2017;377(1):62-70. doi: 10.1056/NEJMra1510062 [published Online First: 2017/07/06]
20. CONseNt methods in paediatric Emergency and urgent Care Trials: University of Liverpool; [Available from: https://www.liverpool.ac.uk/psychology-health-and-society/research/connect/ accessed 24 April 2019.
21. Harron K, Woolfall K, Dwan K, et al. Deferred Consent for Randomized Controlled Trials in Emergency Care Settings. *Pediatrics* 2015;136(5):e1316-22. doi: 10.1542/peds.2015-0512 [published Online First: 2015/10/07]
22. Woolfall K, Frith L, Gamble C, et al. How parents and practitioners experience research without prior consent (deferred consent) for emergency research involving children with life threatening conditions: a mixed method study. *BMJ Open* 2015;5(9):e008522. doi: 10.1136/bmjopen-2015-008522 [published Online First: 2015/09/20]
23. Woolfall K, Frith L, Gamble C, et al. How experience makes a difference: practitioners' views on the use of deferred consent in paediatric and neonatal emergency care trials. *BMC Med Ethics* 2013;14:45. doi: 10.1186/1472-6939-14-45 [published Online First: 2013/11/08]
24. O'Hara CB, Canter RR, Mouncey PR, et al. A qualitative feasibility study to inform a randomised controlled trial of fluid bolus therapy in septic shock. *Arch Dis Child* 2018;103(1):28-32. doi: 10.1136/archdischild-2016-312515 [published Online First: 2017/08/30]
25. Peters MJ, Jones GAL, Wiley D, et al. Conservative versus liberal oxygenation targets in critically ill children: the randomised multiple-centre pilot Oxy-PICU trial. *Intensive Care Med* 2018;44(8):1240-48. doi: 10.1007/s00134-018-5232-7 [published Online First: 2018/06/06]
26. Peters MJ, Khan I, Woolfall K, et al. Different temperature thresholds for antipyretic intervention in critically ill children with fever due to infection: the FEVER feasibility RCT.

For peer review only - http://bmjopen.bmj.com/site/about/guidelines.xhtml

Health Technol Assess 2019;23(5):1-148. doi: 10.3310/hta23050 [published Online First: 2019/02/23]
27. van Dijk M, Peters JWB, van Deventer P, et al. The COMFORT Behavior Scale: a tool for assessing pain and sedation in infants. *The American journal of nursing* 2005;105(1):33-36. doi: 10.1097/00000446-200501000-00019
28. Carter MC, Miles MS. The Parental Stressor Scale: Pediatric Intensive Care Unit. *Maternal-child nursing journal* 1989;18(3):187-98.
29. Varni JW, Seid M, Rode CA. The PedsQL: measurement model for the pediatric quality of life inventory. *Med Care* 1999;37(2):126-39. [published Online First: 1999/02/19]
30. Stevens K. Valuation of the Child Health Utility 9D Index. *PharmacoEconomics* 2012;30(8):729-47. doi: 10.2165/11599120-000000000-00000 [published Online First: 2012/07/14]
31. National Institute for Health and Care Excellence (NICE). Guide to the methods of technology appraisal, 2013.
32. Intensive Care National Audit and Research Centre. FIRST-line support for Assistance in Breathing in Children 2020 [Available from: https://www.icnarc.org/Our-Research/Studies/First-Abc/About2020.
33. Piaggio G, Elbourne DR, Pocock SJ, et al. Reporting of noninferiority and equivalence randomized trials: extension of the CONSORT 2010 statement. *JAMA* 2012;308(24):2594-604. doi: 10.1001/jama.2012.87802
34. Zwarenstein M, Treweek S, Gagnier JJ, et al. Improving the reporting of pragmatic trials: an extension of the CONSORT statement. *BMJ* 2008;337:a2390. doi: 10.1136/bmj.a2390
35. Medical Research Council. Good Research Practice: Principles and Guidelines 2012 [Available from: https://mrc.ukri.org/publications/browse/good-research-practice-principles-and-guidelines/.
36. INTERNATIONAL CONFERENCE ON HARMONISATION OF TECHNICAL REQUIREMENTS FOR REGISTRATION OF PHARMACEUTICALS FOR HUMAN USE. GUIDELINE FOR GOOD CLINICAL PRACTICE. 1996
37. Health Research Authority. UK policy framework for health and social care research. 2017
38. National Institute for Health Research. FIRST-line support for Assistance in Breathing in Children (FIRST-ABC): a master protocol of two randomised trials to evaluate the non-inferiority of high flow nasal cannula therapy versus continuous positive airway pressure for non-invasive respiratory support in paediatric critical care 2019 [Available from: https://www.journalslibrary.nihr.ac.uk/programmes/hta/179428/#/ accessed 5 February 2020.

For peer review only - http://bmjopen.bmj.com/site/about/guidelines.xhtml

Footnotes

Acknowledgements

The authors thank Karen Thomas, Izabella Orzechowska, Michelle Saull and Roger Parslow for their contribution to the set-up and delivery of FIRST-ABC. The authors also thank the research and clinical staff at the participating sites: Addenbrookes' Hospital (Cambridge), Alder Hey Children's Hospital (Liverpool), Birmingham Women and Children's Hospital, Bristol Royal Hospital for Children, Chelsea and Westminster Hospital, Evelina London Children's Hospital, Great North Children's Hospital (Newcastle), Great Ormond Street Hospital (London), Hull Royal Infirmary, James Cook University Hospital (Middlesbrough), John Radcliffe Hospital (Oxford), King's College Hospital (London), Leicester Royal Infirmary and Glenfield Hospital, Noah's Ark Children's Hospital for Wales (Cardiff), Queens Medical Centre (Nottingham), Royal Alexandra Children's Hospital (Brighton), Royal Brompton Hospital (London), Royal Hospital for Sick Children Edinburgh, Royal Manchester Children's Hospital, Sheffield Children's Hospital, Southampton Children's Hospital, St George's Hospital (London), St Mary's Hospital (London) and The Royal London Hospital. The authors acknowledge the UK Paediatric Intensive Care Society – Study Group for supporting the trial.

Contributors

PR is Chief Investigator. ARB is Trial Manager. PR and ARB drafted the manuscript.
PD, RF, RG, DH, JL, KM, PM, MP, KR, ZS, and LT are trial co-applicants and members of the Trial Management Group. LD supported management of the trial. All authors read and approved the final version.

Funding

This trial is funded by the National Institute for Health Research (NIHR) Health Technology Assessment (HTA) Programme (project number: 17/94/28). Great Ormond Street Hospital for Children NHS Foundation Trust is the trial sponsor. The views expressed are those of the author(s) and not necessarily those of the NIHR or the Department of Health and Social Care nor the Sponsor. The funder and sponsor had no role in the writing of this article or the decision to submit the protocol for publication.

Competing interests

None declared.

For peer review only - http://bmjopen.bmj.com/site/about/guidelines.xhtml

Patient consent

Not required.

Ethics approval

The trial has received a favourable ethical opinion from the NHS East of England - Cambridge South Research Ethics Committee (reference number: 19/EE/0185) and approval from the Health Research Authority (Integrated Research Application System (IRAS) number: 260536).

For peer review only

For peer review only - http://bmjopen.bmj.com/site/about/guidelines.xhtml

Figure legends

Figure 1. Algorithm for delivery of High Flow Nasal Cannula.

† Respiratory distress defined as: Mild (one accessory muscle used, mild indrawing of subcostal and intercostal muscles, mild tachypnea, no grunting), Moderate (two accessory muscles used, moderate indrawing of subcostal and intercostal muscles, moderate tachypnea, occasional grunting); or Severe (use of all accessory muscles, severe indrawing of subcostal and intercostal muscles, severe tachypnea, regular grunting).

* Titrate Fi02 while on HFNC to maintain Sp02 ⩾92% (or patient-specific target)

Figure 2. Algorithm for delivery of Continuous Positive Airway Pressure.

† Respiratory distress defined as: Mild (one accessory muscle used, mild indrawing of subcostal and intercostal muscles, mild tachypnea, no grunting), Moderate (two accessory muscles used, moderate indrawing of subcostal and intercostal muscles, moderate tachypnea, occasional grunting); or Severe (use of all accessory muscles, severe indrawing of subcostal and intercostal muscles, severe tachypnea, regular grunting).

* Titrate Fi02 while on HFNC to maintain Sp02 ⩾92% (or patient-specific target)

For peer review only

For peer review only - http://bmjopen.bmj.com/site/about/guidelines.xhtml

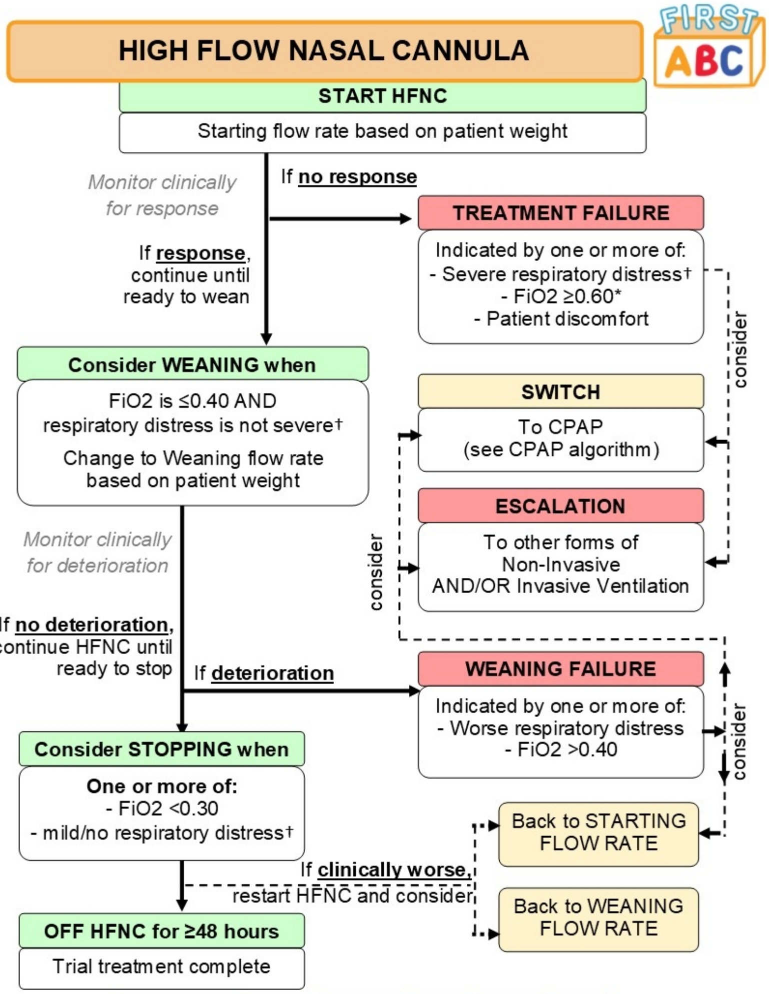

Weight (kg)	≤12	13-15	16-30	31-50	>50
Starting flow rate	2 l/min/kg	25-30 l/min	35 l/min	40 l/min	50 l/min
Weaning flow rate	1 l/min/kg	13-15 l/min	18 l/min	20 l/min	25 l/min

Algorithm for delivery of High Flow Nasal Cannula.

147x209mm (150 x 150 DPI)

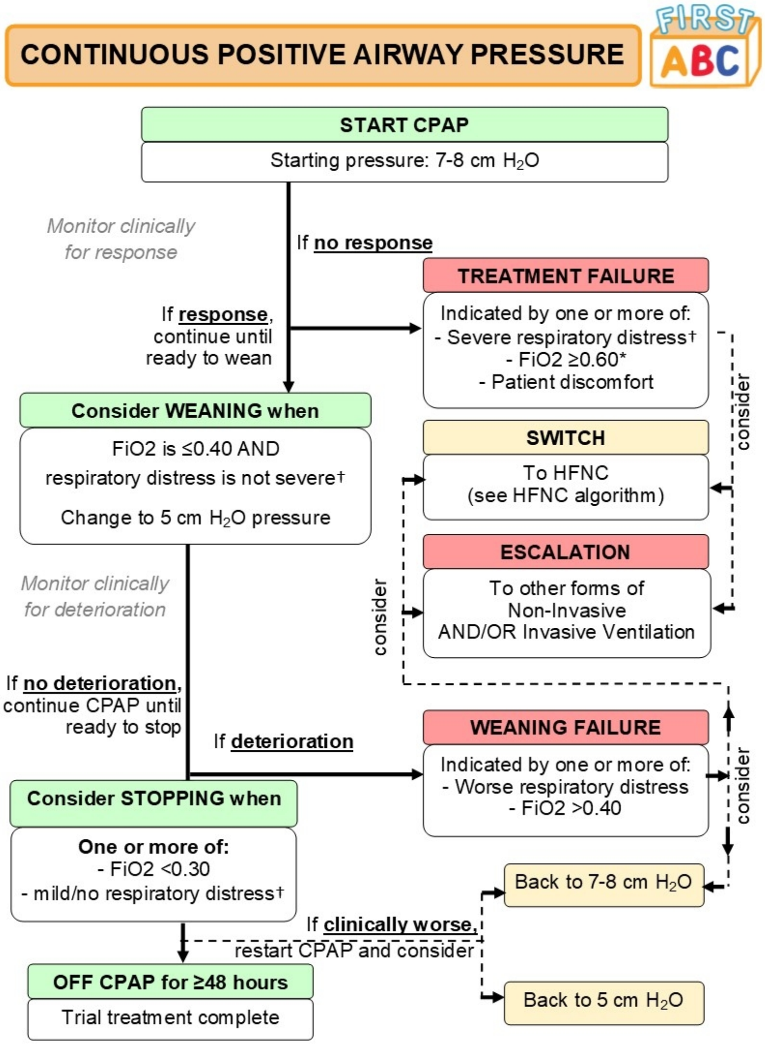

Algorithm for delivery of Continuous Positive Airway Pressure.

147x209mm (150 x 150 DPI)

To be printed on local hospital headed paper

icnarc intensive care national audit & research centre

FIRST ABC

Great Ormond Street Hospital for Children NHS Foundation Trust

FIRST-line support for Assistance in Breathing in Children

Consent Form - Parent or Legal Guardian

Version 1.2, 17 January 2020

To be completed by the Researcher:

Hospital name:	
Trial Number:	
Child's full name:	

To be completed by the Parent or Legal Guardian:

Once you have read and understood each statement – **if you agree, please write your initials in each box**

1.	I confirm that I have read and understood the Participant Information Sheet (version 1.2, dated 27/11/2019) for the above research study. I have had the opportunity to consider the information, ask questions and have had these answered satisfactorily.	
2.	I understand that participation is voluntary, and that I am free to withdraw consent at any time, without giving any reason and without my child's medical care or legal rights being affected.	
3.	I agree to for my child to continue to take part in this study.	
4.	I understand that relevant sections of my child's medical records and data collected during the study (including name, date of birth, postcode and NHS number), held by the NHS or by NHS Digital, may be looked at by individuals from the NHS Trust, the Intensive Care National Audit & Research Centre (ICNARC), NHS Digital or regulatory authorities where it is relevant to my participation in this research. I give permission for these individuals to have access to my child's records.	
5.	I agree to complete a questionnaire about my experiences and reactions to being in the intensive care or high dependency unit.	
6.	I understand that ICNARC will send me a questionnaire to find out how my child is doing in six months time.	
7.	I understand that the information collected in the study will be used to support other research in the future, and may be shared anonymously with other researchers.	
8.	I would like to be contacted about any future related studies.	

Your signature:		Date:	
Your full name (PRINT):			
Researcher signature:		Date:	
Researcher full name (PRINT):			

Once signed please turn over and complete

For peer review only - http://bmjopen.bmj.com/site/about/guidelines.xhtml

FIRST-line support for Assistance in Breathing in Children

Parent or Legal Guardian contact information

To be completed by the Parent or Legal Guardian:

If you would prefer to receive the questionnaire (as detailed on point 6 of the Consent Form) by **email**, please provide your details below:

**Email address:**	
**Telephone number(s):**	

OR

if you would prefer to receive the questionnaire in the **post**, please provide your details below:

**Postal address:**								
**Postcode:**								
**Telephone number(s):**								

1 copy for patient and parent/guardian; 1 copy for Investigator Site File; 1 copy to be kept with hospital notes

For peer review only

For peer review only - http://bmjopen.bmj.com/site/about/guidelines.xhtml

STANDARD PROTOCOL ITEMS: RECOMMENDATIONS FOR INTERVENTIONAL TRIALS

SPIRIT 2013 Checklist: Recommended items to address in a clinical trial protocol and related documents*

Section/item	Item No	Description	Page No.
**Administrative information**			
Title	1	Descriptive title identifying the study design, population, interventions, and, if applicable, trial	P1
Trial registration	2a	Trial identifier and registry name. If not yet registered, name of intended registry	P3
	2b	All items from the World Health Organization Trial Registration Data Set	Throughout
Protocol version	3	Date and version identifier	P20
Funding	4	Sources and types of financial, material, and other support	P24
Roles and responsibilities	5a	Names, affiliations, and roles of protocol contributors	P24
	5b	Name and contact information for the trial sponsor	P19
	5c	Role of study sponsor and funders, if any, in study design; collection, management, analysis, and interpretation of data; writing of the report; and the decision to submit the report for publication, including whether they will have ultimate authority over any of [illegible]	P24
	5d	Composition, roles, and responsibilities of the coordinating centre, steering committee, endpoint adjudication committee, data management team, and other individuals or groups overseeing the trial, if applicable (see Item 21a for data monitoring committee)	P19
**Introduction**			
Background and rationale	6a	Description of research question and justification for undertaking the trial, including summary of relevant studies (published and unpublished) examining benefits and harms for each intervention	P4
	6b	Explanation for choice of comparators	P4
Objectives	7	Specific objectives or hypotheses	P5

For peer review only

Trial design	8	Description of trial design including type of trial (eg, parallel group, crossover, factorial, single group), allocation ratio, and framework (eg, superiority,	P5

For peer review only

For peer review only - http://bmjopen.bmj.com/site/about/guidelines.xhtml

Methods: Participants, interventions, and outcomes

Study setting	9	Description of study settings (eg, community clinic, academic hospital) and list of countries where data will be collected. Reference to where list of study sites can be obtained	P5
Eligibility criteria	10	Inclusion and exclusion criteria for participants. If applicable, eligibility criteria for study centres and individuals who will perform the interventions (eg, surgeons, psychotherapists)	P6
Interventions	11a	Interventions for each group with sufficient detail to allow replication, including how and when they will be administered	P7-10
	11b	Criteria for discontinuing or modifying allocated interventions for a given trial participant (eg, drug dose change in response to harms, participant request, or improving/worsening disease)	P9
	11c	Strategies to improve adherence to intervention protocols, and any procedures for monitoring adherence (eg, drug tablet return, laboratory tests)	NA
	11d	Relevant concomitant care and interventions that are permitted or prohibited during the trial	NA
Outcomes	12	Primary, secondary, and other outcomes, including the specific measurement variable (eg, systolic blood pressure), analysis metric (eg, change from baseline, final value, time to event), method of aggregation (eg, median, proportion), and time point for each outcome. Explanation of the clinical relevance of chosen efficacy and harm outcomes is strongly recommended	P13
Participant timeline	13	Time schedule of enrolment, interventions (including any run-ins and washouts), assessments, and visits for participants. A schematic diagram is highly recommended (see Figure)	P7-14
Sample size	14	Estimated number of participants needed to achieve study objectives and how it was determined, including clinical and statistical assumptions supporting any sample size calculations	P15-16
Recruitment	15	Strategies for achieving adequate participant enrolment to reach target sample size	P7

Methods: Assignment of interventions (for controlled trials)

Allocation:

For peer review only

Sequence generation	16a	Method of generating the allocation sequence (eg, computer- generated random numbers), and list of any factors for stratification. To reduce predictability of a random sequence, details of any planned restriction (eg, blocking) should be provided in a separate document that is unavailable to those who enrol participants or assign	P6-7

For peer review only

For peer review only - http://bmjopen.bmj.com/site/about/guidelines.xhtml

Allocation concealment mechanism	16b	Mechanism of implementing the allocation sequence (eg, central telephone; sequentially numbered, opaque, sealed envelopes), describing any steps to conceal the sequence until interventions are assigned	P6-7
Implementation	16c	Who will generate the allocation sequence, who will enrol participants, and who will assign participants to interventions	P6-7
Blinding (masking)	17a	Who will be blinded after assignment to interventions (eg, trial participants, care providers, outcome assessors, data analysts), and how	NA
	17b	If blinded, circumstances under which unblinding is permissible, and procedure for revealing a participant's allocated intervention during the trial	NA
**Methods: Data collection, management, and analysis**			
Data collection methods	18a	Plans for assessment and collection of outcome, baseline, and other trial data, including any related processes to promote data quality (eg, duplicate measurements, training of assessors) and a description of study instruments (eg, questionnaires, laboratory tests) along with their reliability and validity, if known. Reference to where data collection forms can be found, if not in the protocol	P14-15
	18b	Plans to promote participant retention and complete follow-up, including list of any outcome data to be collected for participants who discontinue or deviate from intervention protocols	P14
Data management	19	Plans for data entry, coding, security, and storage, including any related processes to promote data quality (eg, double data entry; range checks for data values). Reference to where details of data management procedures can be found, if not in the protocol	P15
Statistical methods	20a	Statistical methods for analysing primary and secondary outcomes. Reference to where other details of the statistical analysis plan can be found, if not in the protocol	P16-18
	20b	Methods for any additional analyses (eg, subgroup and adjusted analyses)	P17-18
	20c	Definition of analysis population relating to protocol non-adherence (eg, as randomised analysis), and any statistical methods to handle missing data (eg, multiple imputation)	P16

For peer review only

Methods: Monitoring

Data monitoring	21a	Composition of data monitoring committee (DMC); summary of its role and reporting structure; statement of whether it is independent from the sponsor and competing interests; and reference to where further details about its charter can be found, if not in the	P19

For peer review only

	21b	Description of any interim analyses and stopping guidelines, including who will have access to these interim results and make the final decision to terminate the trial	P17-18
Harms	22	Plans for collecting, assessing, reporting, and managing solicited and spontaneously reported adverse events and other unintended effects of trial interventions or trial conduct	P12-13
Auditing	23	Frequency and procedures for auditing trial conduct, if any, and whether the process will be independent from investigators and the sponsor	P19
**Ethics and dissemination**			
Research ethics approval	24	Plans for seeking research ethics committee/institutional review board (REC/IRB) approval	P19,25
Protocol amendments	25	Plans for communicating important protocol modifications (eg, changes to eligibility criteria, outcomes, analyses) to relevant parties (eg, investigators, REC/IRBs, trial participants, trial registries, journals, regulators)	Not provided
Consent or assent	26a	Who will obtain informed consent or assent from potential trial participants or authorised surrogates, and how (see Item 32)	P11-12
	26b	Additional consent provisions for collection and use of participant data and biological specimens in ancillary studies, if applicable	NA
Confidentiality	27	How personal information about potential and enrolled participants will be collected, shared, and maintained in order to protect confidentiality before, during, and after the trial	P19
Declaration of interests	28	Financial and other competing interests for principal investigators for the overall trial and each study site	P24
Access to data	29	Statement of who will have access to the final trial dataset, and disclosure of contractual agreements that limit such access for investigators	P19
Ancillary and post-trial care	30	Provisions, if any, for ancillary and post-trial care, and for compensation to those who suffer harm from trial participation	NA
Dissemination policy	31a	Plans for investigators and sponsor to communicate trial results to participants, healthcare professionals, the public, and other relevant groups (eg, via publication, reporting in results databases, or other data sharing arrangements), including any publication restrictions	P20

For peer review only

31b	Authorship eligibility guidelines and any intended use of professional writers	Not provided
31c	Plans, if any, for granting public access to the full protocol, participant- level dataset, and statistical code	Full protocol is referenced

For peer review only

**Appendices**			
Informed consent materials	32	Model consent form and other related documentation given to participants and authorised surrogates	Supplementary file
Biological specimens	33	Plans for collection, laboratory evaluation, and storage of biological specimens for genetic or molecular analysis in the current trial and for [illegible]	NA

*It is strongly recommended that this checklist be read in conjunction with the SPIRIT 2013 Explanation & Elaboration for important clarification on the items. Amendments to the protocol should be tracked and dated. The SPIRIT checklist is copyrighted by the SPIRIT Group under the Creative Commons "Attribution-NonCommercial-NoDerivs 3.0 Unported" license.

For peer review only
